# Polysulfone mixed matrix membranes embedded with sub-25 nm NH_2_-MIL-101(Cr) for enhanced divalent salt separation and permeability

**DOI:** 10.1039/d6ra07963f

**Published:** 2026-10-14

**Authors:** Mubark Alshareef, Ahmed Alharbi, Mohamed S. Fahmi

**Affiliations:** a Department of Chemistry, Faculty of Science, Umm Al-Qura University Makkah Saudi Arabia mmshreef@uqu.edu.sa +00201002535379; b Suez University, Faculty of Science, Chemistry Department 43518 Suez Egypt masf23@sci.suezuni.edu.eg

## Abstract

High-performance nanofiltration (NF) membranes are essential for sustainable water treatment but are often limited by the permeability-fouling trade-off. In this study, highly crystalline sub-25 nm NH_2_-MIL-101(Cr) nanoparticles (19–21 nm), possessing a high BET surface area (1700.18 m^2^ g^−1^) and permanent pores (2.04 nm), were hydrothermally synthesized and incorporated into polysulfone (PSF) mixed matrix membranes *via* phase inversion. The uniform distribution of hydrophilic NH_2_ groups enhanced membrane hydrophilicity, reducing the water contact angle from 76.2 ± 2° to 56.1 ± 2° at the optimum loading of 0.75 wt%. At 5 bar using a 2000 ppm feed solution, the optimized membrane achieved a water flux of 15.3 kg m^−2^ h^−1^, representing a 2.3-fold increase over pristine PSF, while maintaining high rejection of MgSO_4_ (86.5%) and CaSO_4_ (96.2%) through combined steric hindrance and Donnan exclusion mechanisms. Flux increased linearly with pressure up to 17.8 kg m^−2^ h^−1^ at 7.0 bar. Organic fouling tests with 10 ppm humic acid demonstrated excellent antifouling performance, with flux recovery ratios of 83.2% and 68.5% after the first and second alkaline cleaning cycles, respectively. These findings demonstrate that the PSF/NH_2_-MIL-101(Cr) mixed matrix membrane effectively overcomes the permeability-fouling trade-off, offering a promising platform for high-performance and long-term desalination applications.

## Abstract 2

Sub-25 nm NH2-MIL-101(Cr) nanoparticles embedded in polysulfone membranes boost water flux 2.3-fold (15.3 kg m^−2^ h^−1^) while achieving 86.5% MgSO_4_ and 96.2% CaSO_4_ rejection, breaking the permeability–selectivity trade-off.

## Graphical abstract

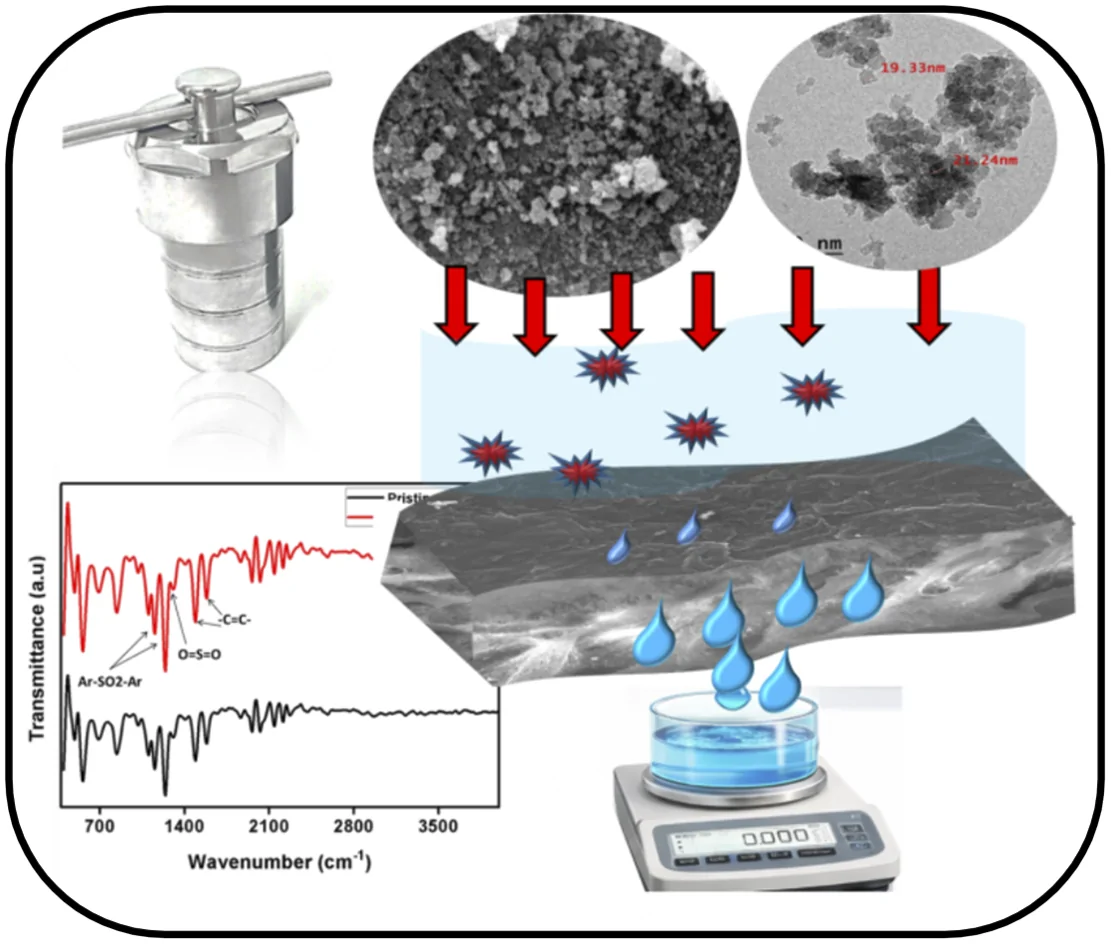

## 1 Introduction

Water desalination represents one of the most critical global challenges of the twenty-first century, playing a pivotal role in the production of high-quality reusable water and the effective removal of persistent contaminants from wastewater streams.^1^ With the continuous growth in global water demand and the rapid depletion of freshwater resources, the development of advanced desalination technologies has become an urgent necessity to meet the needs of expanding populations and increasing industrial activities. However, the large-scale application of membrane-based technologies is often constrained by limited productivity resulting from membrane fouling phenomena.^1,2^ Membrane fouling arises from the deposition and accumulation of rejected particles or compounds on the membrane surface, leading to increased operational and maintenance costs, reduced water recovery, deterioration of permeate quality, shortened operational lifespan, and premature membrane replacement.^2^ Polysulfone (PSF) membranes are among the most widely used polymeric materials in membrane separation technologies due to their excellent mechanical strength, high chemical resistance, and good thermal stability.^3^ Nevertheless, polysulfone, along with polyethersulfone (PES) and polyvinylidenefluoride (PVDF), is classified as a slightly hydrophobic polymer.^3^ The static water contact angle of these polymers typically ranges between 70° and 80°, rendering them particularly susceptible to fouling.^3^ This inherent hydrophobicity promotes the adsorption of organic and biological substances onto the membrane surface, thereby reducing separation efficiency and increasing long-term operational costs.

In recent years, metal–organic frameworks (MOFs) have emerged as highly promising materials for enhancing the performance of polymeric membranes.^4,5^ MOFs are three-dimensional porous crystalline materials composed of metal ions or clusters coordinated with organic ligands, forming periodic network structures.^4,5^ These materials are distinguished by exceptionally high specific surface areas, tunable porosity and topology, and remarkable structural versatility, making them attractive candidates for the fabrication of high-performance nanocomposite membranes.^4^ Unlike conventional inorganic fillers, MOFs exhibit greater structural flexibility due to their hybrid organic–inorganic nature, allowing precise control over pore size, framework geometry, and physicochemical properties.^6^ Such tunability enables MOFs to impart enhanced membrane permeability and selectivity.^4^ To date, more than 84 000 different MOF structures have been reported, offering virtually unlimited possibilities in terms of pore architecture, functionality, and application potential.^5^ The incorporation of MOFs into polymeric membranes, including polysulfone-based systems, has gained significant attention owing to their adjustable porosity, excellent selectivity, high permeability, and improved compatibility with polymer matrices.^7^ MOF integration confers several advantageous features, such as enhanced hydrophilicity, photocatalytic activity, antifouling behavior, and self-cleaning capability, which are highly beneficial for water and wastewater treatment applications.^4,7^ This hybridization strategy effectively addresses the intrinsic limitations of polymeric membranes, particularly their hydrophobicity and fouling propensity.^3,6^ For instance, the incorporation of ZIF-8 into PES membranes has been reported to reduce the water contact angle from 77° to 62°, while UiO-66 demonstrates outstanding hydrophilicity enhancement.^10^

Nanofiltration (NF) is a pressure-driven membrane technology in which MOF-incorporated membranes have been extensively applied for water and wastewater treatment.^1^ NF membranes are capable of selectively rejecting multivalent salts, low-molecular-weight organic compounds, and emerging micropollutants at significantly lower operating pressures compared to reverse osmosis and consists from simple compartment as depicted in Fig. 1.^8^ MOF-based hybrid membranes have been developed to improve the removal efficiency of hazardous contaminants, including persistent organic pollutants (POPs) and heavy metals from wastewater streams.^8,9^ Several studies have demonstrated that MOF-modified membranes can achieve contaminant removal efficiencies exceeding 95% for organic pollutants and nearly complete removal of suspended solids.^1^ From a physicochemical perspective, MOFs significantly enhance membrane hydrophilicity, which is a key factor in mitigating membrane fouling.^10,11^ For example, ZIF-8 incorporation has been shown to increase water flux by more than 2.5 times while maintaining up to 90% flux recovery after multiple filtration cycles.^11^ MOFs introduce additional water transport pathways within the membrane matrix, facilitating water penetration and increasing overall permeability.^12,13^ In terms of solute rejection, MOFs improve separation performance through combined mechanisms of molecular sieving, electrostatic exclusion, and adsorption.^14,15^ Their tunable pore structures enable selective separation of target contaminants while maintaining high water flux.

**Fig. 1:**
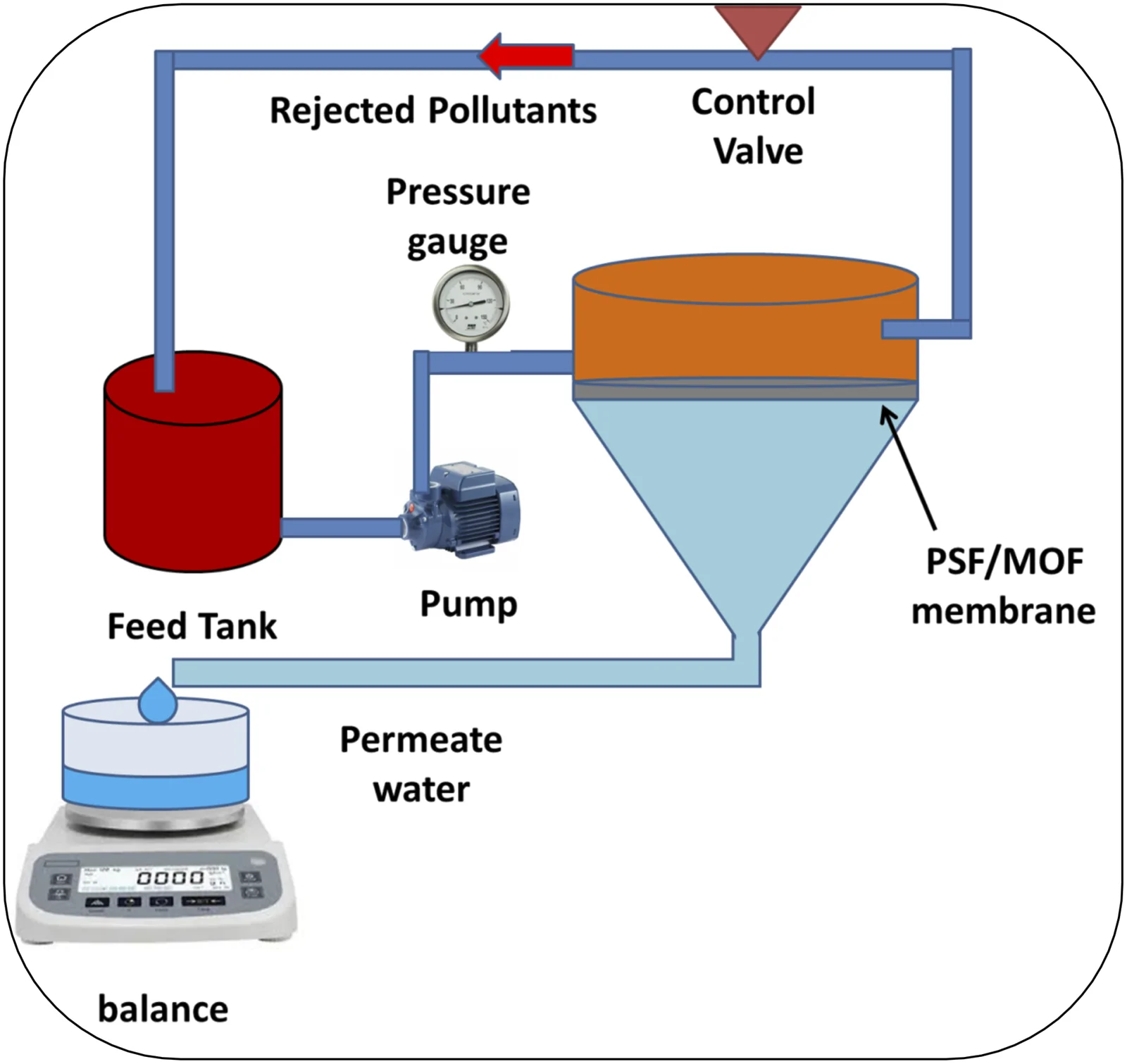
Schematic diagram of the nanofiltration system.

Beyond separation performance, MOF-incorporated membranes exhibit unique functional advantages, particularly in photocatalytic and self-cleaning applications.^16,17^ Certain MOFs, such as Ti-based MOFs and MIL-88B(Fe), display photocatalytic activity capable of degrading organic pollutants under light irradiation.^16,18^ This functionality enables *in situ* self-cleaning of membranes and substantially reduces fouling accumulation. Moreover, some MOFs possess intrinsic antibacterial properties, which help suppress biofouling.^19,20^ For example, Ag-based MOF membranes have demonstrated excellent antibacterial activity against both Gram-positive and Gram-negative bacteria. From a mechanical standpoint, the incorporation of MOFs can enhance the mechanical strength and thermal stability of polymeric membranes.^21,22^ MOF particles act as nanoscale reinforcing agents, improving the mechanical integrity of the polymer matrix. Additionally, MOFs such as UiO-66 and ZIF-8 exhibit exceptional chemical and thermal stability, contributing to prolonged membrane lifespan.^23,24^ This improved stability enables membrane operation under harsh conditions, including wide pH ranges and elevated temperatures.

MOF-incorporated membranes have demonstrated broad applicability in desalination and wastewater treatment processes. In nanofiltration applications, these membranes exhibit high efficiency in removing multivalent salts, organic dyes, and heavy metals.^25,26^ For instance, MIL-101(Cr)-based membranes have shown excellent stability and performance in water treatment applications.^27^ In forward osmosis and membrane distillation processes, MOFs contribute to enhanced flux and selectivity while effectively mitigating fouling.^28,29^ Additionally, MOF-modified membranes have been successfully employed in oil–water emulsion separation and the removal of persistent organic contaminants.^30^ Despite their advantages, the large-scale application of MOF-incorporated membranes faces challenges, including poor compatibility and dispersion of MOF particles, which can cause defects and reduced performance. Long-term stability in aqueous environments, particularly under extreme pH or high humidity, is also a concern, making water-stable MOFs like UiO-66 and ZIF-8 essential. Economic feasibility and scalability are additional issues, as high-quality MOF synthesis is costly and uniform incorporation into membranes is technically demanding. Future research should focus on multifunctional MOFs with photocatalytic and antibacterial properties, novel water-stable MOFs, innovative fabrication strategies (*e.g.*, 3D printing, self-assembly), integration with other nanomaterials for hybrid membranes, and computational modeling for optimal design. Overall, PSF membranes modified with MOFs present a promising approach for high-performance nanofiltration, offering enhanced permeability, selectivity, and multifunctionality to address water scarcity and support sustainable water management.

## 2 Materials and methods

### 2.1. Materials

Polysulfone (PSF, Udel P-3500) pellets used as the primary host polymer matrix were supplied by Solvay Co. *N*,*N*-dimethylformamide (DMF, ≥99.5%) utilized as the organic solvent for dope solutions was purchased from Fisher Chemical. For the hydrothermal synthesis of the amine-functionalized metal–organic framework (NH_2_-MIL-101(Cr)), chromium nitrate nonahydrate (Cr(NO_3_)_3_·9H_2_O), 2-aminoterephthalic acid (H_2_ATA), and hydrofluoric acid (HF) were purchased from Sigma-Aldrich. PEG was purchased from SRL, Acetone, Ethyl alcohol, and sodium chloride (NaCl) were provided by ADWIC Co. All chemical reagents and analytical solvents were utilized as received without further post-synthetic purification steps. Locally sourced deionized (DI) water was strictly utilized as the non-solvent coagulation bath medium and for post-fabrication membrane washing protocols. Flat, high-smoothness glass plates (25 cm × 28 cm × 4 mm) and a precision cylindrical steel casting rod (length: 40 cm, diameter: 10 mm, equipped with a fixed groove depth of ∼120 µm) were obtained from the local commercial market for control of film thickness during the phase inversion casting sequences.

### 2.2. Synthesis of NH_2_-MIL-101(Cr)

NH2-MIL-101(Cr) was synthesized using a modified hydrothermal method. Chromium nitrate nonahydrate (Cr(NO_3_)_3_·9H_2_O, 1.63 g), 2-aminoterephthalic acid (0.89 g), and hydrofluoric acid (HF, 0.16 g) were dissolved in distilled water (20 mL) and stirred for 30 minutes to form a homogeneous mixture. The resulting solution was transferred to a Teflon-lined stainless-steel autoclave and sealed. The autoclave was heated at 220 °C for 8 hours under static conditions. After cooling to room temperature, the obtained green solid was collected by centrifugation, thoroughly washed with purified water and acetone, and dried under vacuum overnight. The as-synthesized product was finally activated at 160 °C under vacuum overnight to yield NH_2_-MIL-101(Cr).

### 2.3. Preparation of NH_2_-MIL-101(Cr)/PSF composite membranes

PSF/NH_2_-MIL-101(Cr) (PSF/M101-Cr) composite membranes were prepared *via* the wet phase inversion technique using DMF as the solvent. The casting solution was formulated with a PSF concentration 15% (wt/v), where 15 g of PSF for 85 mL of DMF which was used as the solvent. The polymer was dissolved (3.0 g) in DMF (17 mL) under continuous magnetic stirring at 60 °C until a clear and homogeneous solution was obtained. NH_2_-MIL-101(Cr) was incorporated into the PSF solution at loadings of 0.5, 0.75, 1.0, and 1.25% (wt/wt) with respect to the polymer content, corresponding to MOF concentrations of approximately 0.075, 0.1125, 0.150, and 0.1875 g dL^−1^ respectively. Prior to incorporation, the required amount of NH_2_-MIL-101(Cr) was dispersed in a calculated amount of total DMF volume and ultrasonicated for 30 min to ensure uniform dispersion and prevent particle agglomeration. The MOF suspension was then slowly added to the PSF solution under continuous stirring and mixed for 12 h to obtain a stable and homogeneous dope solution. The resulting casting solutions were degassed to remove trapped air bubbles and subsequently cast onto a clean glass plate using a casting knife rod with controlled thickness. Immediately after casting, the films were immersed in a DI water coagulation bath at room temperature (25 ± 2 °C) to induce phase inversion. After complete solvent–nonsolvent Ions exchange, the formed membranes were thoroughly washed and stored in DI water prior to characterization and evaluation with NF unit.

### 2.4. Nanofiltration system setup

The nanofiltration performance, including hydraulic permeability and salt rejection efficiency, was systematically evaluated using a laboratory-scale cross-flow filtration system customized for flat-sheet configurations, as illustrated in Fig. 1. The filtration cell consists of a high-pressure module divided into two symmetric compartments separated by the fabricated mixed matrix membrane, providing an effective filtration area of 7.0 cm^2^. The feed solution was continuously circulated from a 1 L polyethylene feed reservoir into the upper compartment of the module utilizing a precision miniature high-pressure hydraulic pump with an operational power capacity 72 watt, max pressure up to of 1 MPa and a max flow rate of 6 L min^−1^. To ensure strict hydrodynamic control, an inline analog pressure gauge was integrated directly through the feed circle inlet to monitor the transmembrane pressure (TMP). Concurrently, the lower permeate compartment of the cell features an engineered conical geometry designed to eliminate dead volume and streamline hydraulic flow. The purified permeate stream exits the conical base and is collected in a receiving beaker positioned on a high-precision digital analytical balance to monitor the real-time weight changes, which were subsequently used to calculate the volumetric permeate flux (*J*, kg m^−2^ h^−1^) from eqn (1).1
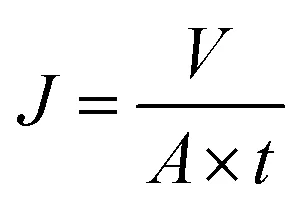
where *J* refers to Flux (kg m^−2^ h^−1^), (*V*) volume of permeate (m^3^) in (*t*) time (h) on (*A*) membrane area (m^2^).

Furthermore, the total dissolved solids (TDS) concentrations of both the bulk feed solution and the collected permeate streams were periodically determined using a calibrated digital conductivity meter to compute the exact salt rejection percentage (S.R%) from eqn (2).2
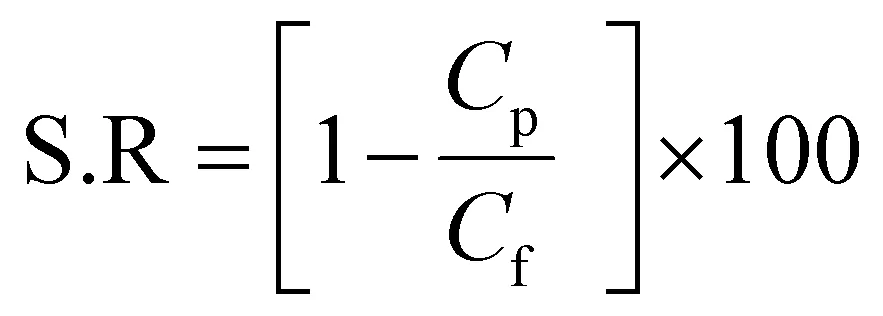
where *C*_f_ represents feed concentration reservoir and *C*_p_ refers to permeate water concentration.

All filtration experiments were performed at a stabilized ambient temperature (25 ± 1 °C) under an operating pressure ranging from 1 to 7.0 bars, and the average values were reported from three independent membrane coupons to guarantee statistical reproducibility.

The molecular weight cut-off (MWCO) of the optimized membrane was determined from its corresponding MWCO curve, obtained by plotting the rejection percentage of the PEG solutes against their respective molecular weights, with the MWCO defined as the molecular weight corresponding to 90% solute rejection. Based on this rejection profile, the mean effective pore diameter (dp) of the optimized membrane was further estimated using the following empirical relationship:^31^

3 dp = 33.46 × 10^−3^ × *M*^0.557^

where *M* denotes the molecular weight of the PEG solute (Da) and dp represents the corresponding mean pore diameter (nm) of the optimized membrane. The Instrumental characterization details were depicted in SI file.

## 3 Results and discussion

### 3.1. Characterization

#### 3.1.1. Morphological characterization of NH_2_-MIL-101(Cr) nanofiller

The morphology and surface characterization of the hydrothermally synthesized NH_2_-MIL-101(Cr) nanoparticles were investigated utilizing integrated SEM and TEM, to evaluate their structural compatibility as functional nano-fillers within PSF-based hybrid matrices as depicted in Fig. 2. The SEM micrographs (Fig. 1a and b) reveal the formation of irregular, hierarchically aggregated architectures composed of highly interconnected nano-sized MOF crystallites. These micro-scale agglomerates exhibit a highly porous and rough surface texture, which is a characteristic thermodynamic footprint of MIL-101-type octahedral frameworks generated under pressurized hydrothermal conditions.^5^ This hierarchical, granular morphology offers abundant interparticle voids and open, unhindered porous domains that are anticipated to act as extra-framework channels for accelerated molecular water transport when embedded into the host polymer network.^13^ Furthermore, the prominent surface corrugation is structurally beneficial for enhancing the mechanical interfacial adhesion and steric interlocking between the crystalline MOF boundaries and the amorphous PSF chains, thereby suppressing macro-phase separation and minimizing the formation of non-selective interfacial void defects.^16^ The TEM micrographs (Fig. 2c and d) further substantiate the discrete nanoscale nature of the synthesized framework, manifesting quasi-spherical nanoparticles tightly assembled into clusters with a narrow size distribution localized between 19 and 21 nm. This ultra-small, controlled grain size is highly advantageous for advanced nanofiltration engineering, as it guarantees a homogeneous, atomic-scale dispersion of the filler within the thin skin layer of the membrane without disrupting the intrinsic structural integrity of the polymer matrix.^10,26^ Mechanistically, the integration of these highly crystalline, sub-25 nm nodes introduces a dual-transport mechanism; it leverages the intrinsic microporous cages of the MIL-101 framework to serve as water-selective molecular sieves while simultaneously creating shortened tortuosity paths and localized water pathways that significantly decrease the hydraulic diffusion length.^17^ Additionally, the heavy chemical decoration of the internal and external framework walls with primary amine (–NH_2_) functional groups induces a strong localized dipole moment, which promotes excellent surface wettability and facilitates fast hydrogen-bonding cascades (O–H⋯N–H) with passing water molecules, fundamentally boosting the boundary water sorption kinetics.^14,30^

**Fig. 2:**
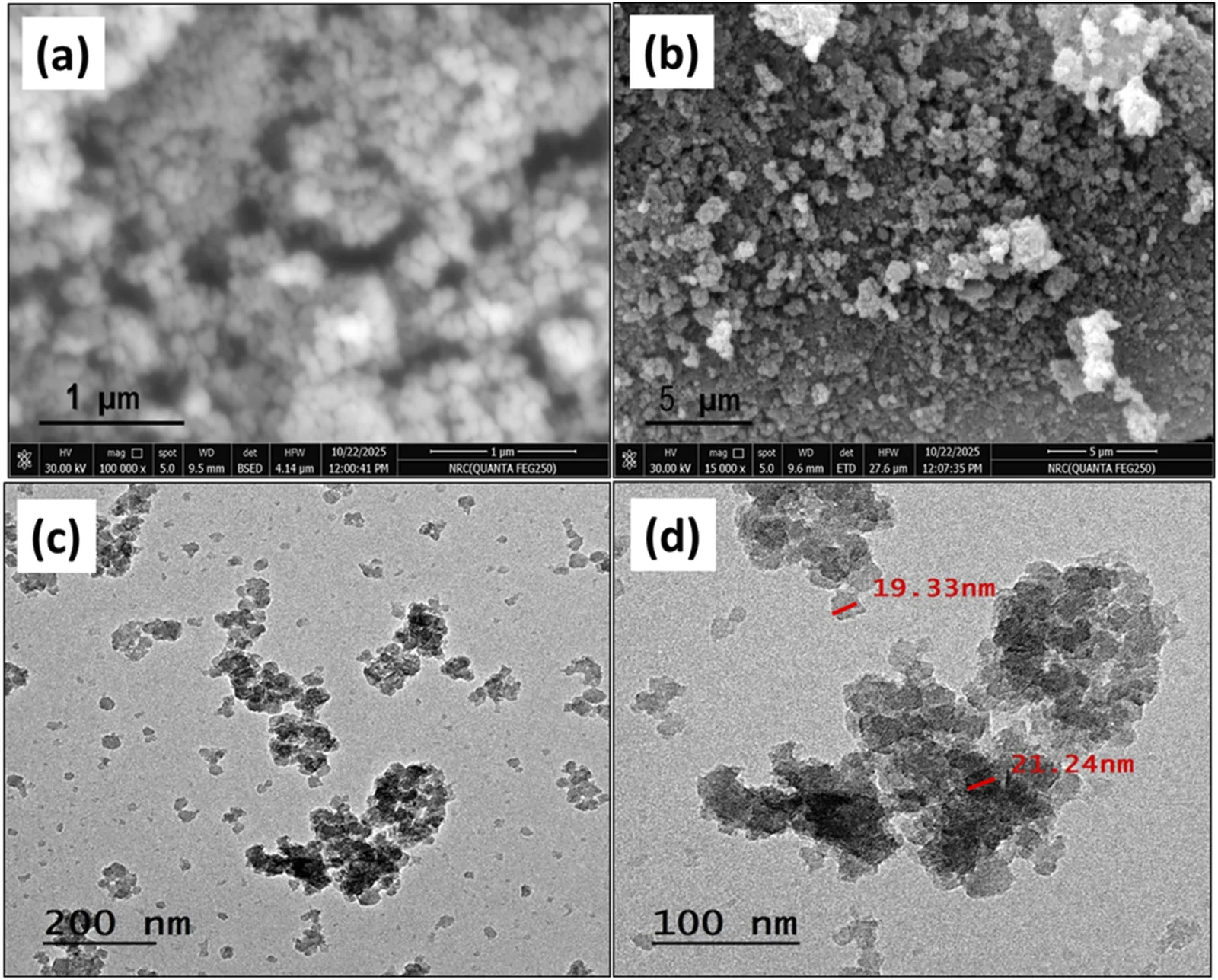
SEM images (a and b) with magnification 100 000× and 15 000× respectively and accelerating voltage 30 kv, TEM images (c and d), of NH_2_-MIL-101(Cr) showing its hierarchical porous morphology and nanoscale particle size (∼19–21 nm).

#### 3.1.2. Textural properties and BET surface area analysis of NH_2_-MIL-101(Cr)

The nitrogen adsorption–desorption isotherm and the corresponding pore size distribution of the hydrothermally synthesized NH_2_-MIL-101(Cr) nanoparticles, providing deep insights into their textural benchmarks and structural porosity, as depicted in Fig. 3. The sorption profile exhibits a hybrid Type IV isotherm standard with a well-defined H1 hysteresis loop at a relative pressure (*P*/*P*_0_) range of 0.4–0.9, representing a hierarchical porous network containing both micro- and mesoporous domains. The initial steep nitrogen uptake at ultralow relative pressures (*P*/*P*_0_ < 0.1) is driven by the rapid micropore filling within the crystalline cages of the MIL-101 architecture.^5^ Concurrently, the subsequent multi-layer adsorption and the prominent hysteresis loop at elevated pressures signify capillary condensation taking place within the mesoporous domains, which arise from the hierarchical interparticle voids generated during the self-assembly of the nanocrystallites, perfectly corroborating the structural aggregates observed in the SEM and TEM analysis.^13,17^ Quantitative evaluation of the textural isotherms reveals an exceptional BET specific surface area of 1700.18 m^2^ g^−1^, paired with a total pore volume of 0.87 cm^3^ g^−1^ and an average pore diameter of 2.04 nm. This remarkably high surface area and large pore volume are consistent with successfully activated amine-functionalized chromium frameworks reported in high-tier literature, where the framework shrinkage or pore blockage by residual unreacted amino-terephthalic acid linkers is effectively minimized through optimized vacuum activation at 160 °C.^12,14^ In the context of membrane separation engineering, this ultra-high surface area paired with a pore diameter of 2.04 nm delivers a strategic dual-advantage; it exposes a massive density of hydrophilic primary amine (NH_2_) active sites to pass target streams, while the intrinsic porous channels provide low-resistance molecular sieving conduits that shorten the water diffusion pathways.^21,30^ Consequently, these outstanding textural traits justify the profound hydraulic enhancements, anti-wetting durability, and high salt rejection efficiencies when embedding these nanofiller in small quantities into the host PSF matrix.^3,29^

**Fig. 3:**
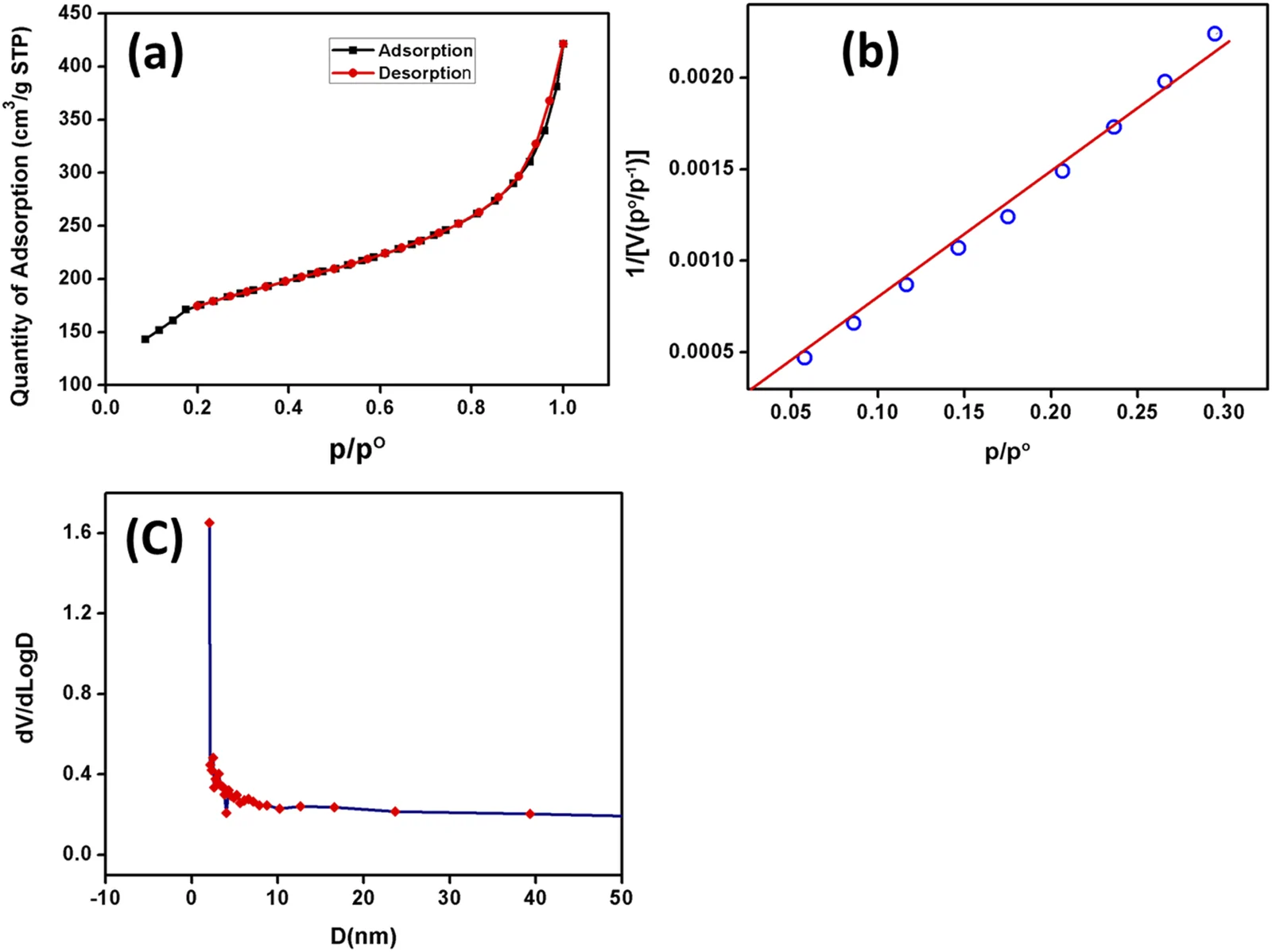
N_2_ adsorption–desorption isoth-erms (a), BET surface area plot (b), and pore size distribution (c) of NH_2_-MIL-101(Cr), confirming its high surface area and hierarchical porous structure suitable for NF membrane applications.

#### 3.1.3. FTIR and XRD of NH_2_-MIL-101(Cr)

The chemical functionalities and crystalline integrity of the hydrothermally synthesized NH_2_-MIL-101(Cr) nanoparticles were rigorously verified using integrated FTIR and XRD analyses, as illustrated in Fig. 4a and b. The FTIR spectrum (Fig. 4a) exhibits the characteristic vibrational bands, confirming the successful formation of the amino-functionalized metal–organic framework. The broad absorption envelope centered at 3361.2 cm^−1^ is assigned to the overlapping stretching vibrations of secondary amino (–NH_2_) and hydroxyl (–OH) groups, indicating the successful incorporation of amino functionalities within the benzene-1,4-dicarboxylic acid core along with hydrogen-bonded adsorbed moisture.^14^ The shoulder band at 2933.8 cm^−1^ corresponds to the symmetric and asymmetric C–H stretching vibrations of the aromatic ring within the organic linkers.^8^ Crucially, the strong, distinct absorptions observed at 1654.24 cm^−1^, 1615 cm^−1^, and 1595 cm^−1^ are attributed to the stretching vibrations of C=O groups from carboxylate moieties coordinated to the metal centers. Specifically, the splitting peaks at 1615 cm^−1^ and 1595 cm^−1^ are indicative of asymmetric COO^−^ stretching, while the sharp band at 1434 cm^−1^ corresponds to the symmetric COO^−^ stretching mode, collectively validating a robust coordination bridge between the terephthalate ligands and the Cr^3+^ clusters.^5,12^ The successful chemical grafting of the amino groups onto the framework is further substantiated by the appearance of the diagnostic bands at 1339 cm^−1^ and 1257.7 cm^−1^, which are characteristically assigned to the aromatic C–N stretching vibrations.^16,30^ Additionally, the finger-print bands located at low frequencies (767.5 cm^−1^ and 661.2 cm^−1^) are associated with the Cr–O stretching and lattice vibrations of the octahedral metal clusters, reflecting successful metal–ligand nucleation networks.^21^

**Fig. 4:**
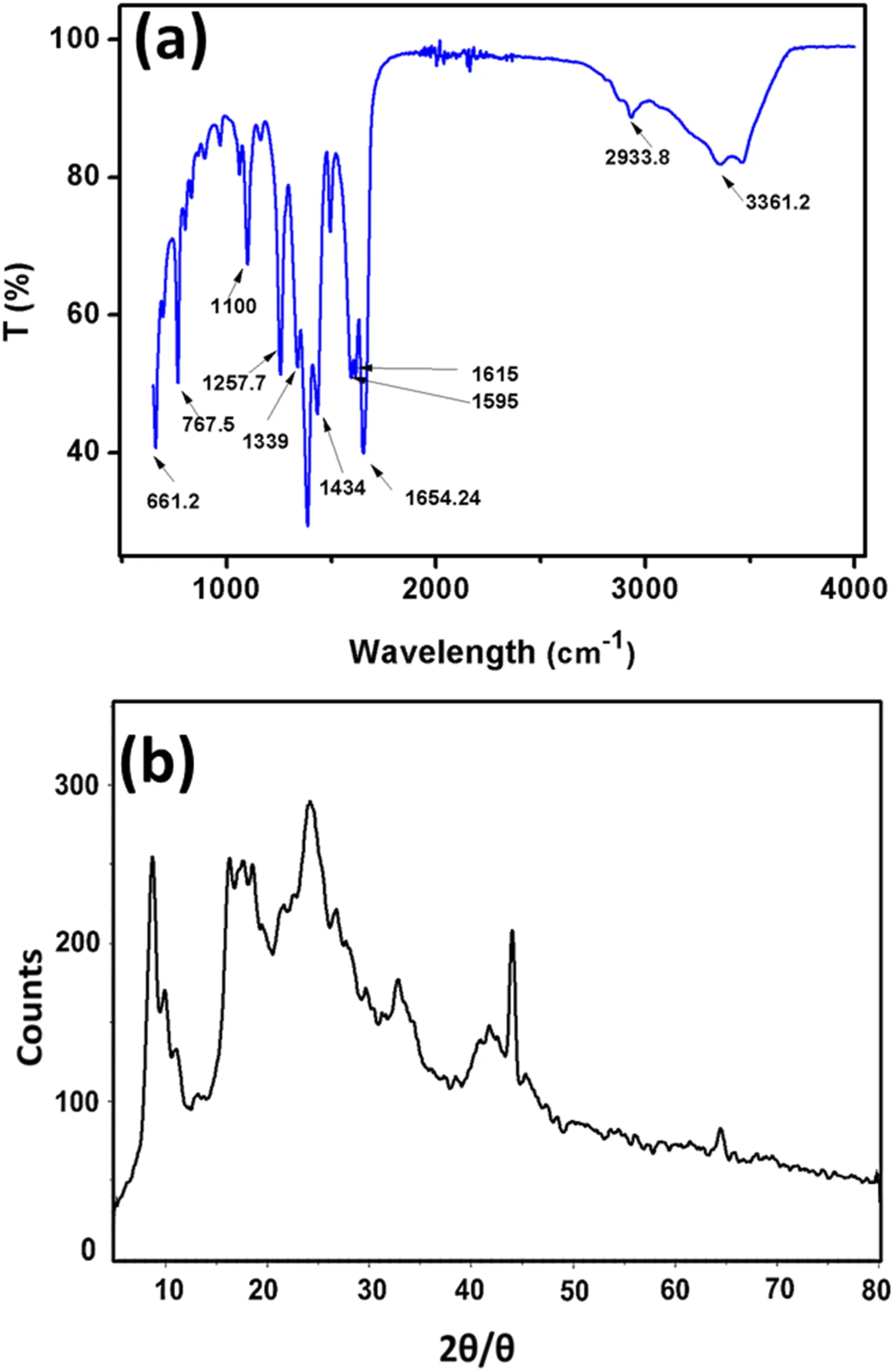
(a) FTIR spectrum and (b) XRD pattern of the synthesized NH_2_-MIL-101(Cr) nanoparticles.

To cross-examine the structural data with the long-range atomic order, the crystalline architecture was evaluated *via* X-ray diffraction, as presented in the XRD pattern (Fig. 4b). The sample exhibits exceptionally sharp, high-intensity diffraction reflections at low 2*θ* angles, with dominant, well-resolved peaks emerging at 3.3°, 5.2°, 5.9°, 8.5°, 9.1°, and 16.5°. These specific angular positions and their sharp line profiles serve as a definitive crystalline fingerprint that matches the simulated cubic crystal system of the octahedral MIL-101 topology (*Fm*-3*m* space group).^4,13^ The preservation of these highly intense, low-angle reflections confirms that the crystalline framework maintained its permanent porosity and structural stability during the post-synthetic purification and vacuum activation protocols, without experiencing pore collapse or skeletal framework shrinkage.^17^ Furthermore, the complete absence of broad amorphous humps or extraneous impurity peaks (such as uncoordinated aminoterephthalic acid or chromium oxide phases) confirms the high phase purity and superior crystallinity of the synthesized nanofiller.^26^ When incorporated into the PSF matrix, this highly crystalline and chemically functionalized NH_2_-MIL-101(Cr) structural network operates as a robust, non-deformable filler. The open microporous windows verified by XRD, paired with the hydrophilic –NH_2_ sites proven by FTIR, work in synergy to establish unhindered water-permeable conduits that drastically lower hydraulic resistance and promote outstanding nanofiltration flux and selective salt retention performance.^3,29^

#### 3.1.4. Morphology investigation of PSF/MIL-101(Cr) membranes

The surface topographies and cross-sectional microstructures of both the pristine PSF and the optimized PSF/MIL-101(Cr) mixed-matrix membranes 0.75 wt% evaluated *via* SEM as illustrated in Fig. 5. The surface view of the pristine membrane (Fig. 5a) exhibits a relatively smooth, dense, and uniform skin layer with isolated, small pore openings, while its corresponding cross-section (Fig. 5b) displays a conventional asymmetric morphology consisting of a thin top-dense selective layer supported by a underlying spongy-like substructure layer. On the Other hand, the incorporation of 0.75 wt% hydrophilic NH_2_-MIL-101(Cr) nanoparticles profoundly alters the membrane architecture; the top surface (Fig. 5c) reveals a highly rough surface with a significant increase in surface roughness and the proliferation of structural wrinkles on the modified membrane skin signify a substantial expansion in the effective surface area, where the well-dispersed MOF crystals are integrated into the polymer matrix without visible particle agglomeration. Crucially, the cross-sectional profile of the hybrid membrane (Fig. 5d) undergoes a complete transformation, exhibiting a highly developed, wide, and continuous finger-like macrovoid configuration that extends vertically from the top skin layer down to the bottom porous support. The fundamental thermodynamic and kinetic mechanism driving this structural evolution is governed by the altered phase separation kinetics during the non-solvent induced phase separation (NIPS) process; the presence of the hydrophilic NH_2_-MIL-101(Cr) nanoparticles, which possess abundant hydrophilic primary amine NH_2_ and hydroxyl decoration, severely decreases the thermodynamic stability of the DMF casting dope solution, thereby inducing a rapid, instantaneous demixing between the solvent (DMF) and the non-solvent (DI water) in the coagulation bath.^8,12^ This accelerated out-flux of DMF paired with the fast localized hydro-penetration forces the polymer chains to solidify rapidly around the hydro-active MOF nuclei, encouraging the growth of highly interconnected, open finger-like channels rather than slow, delayed demixing symmetric structures.^4,16^ Hydrodynamically, this engineered macrovoid structure drastically suppresses the internal transport resistance against permeating water molecules.^13,29^ Furthermore, the highly open channel network and the uniform distribution of the crystalline, porous MOF channels act as rapid water-selective pathways that preferentially absorb and transport water molecules while maintaining high structural rejections against divalent salts *via* steric and electrostatic barriers, establishing this hybrid morphology as an ideal matrix for high-flux desalination applications.^20,28^

**Fig. 5:**
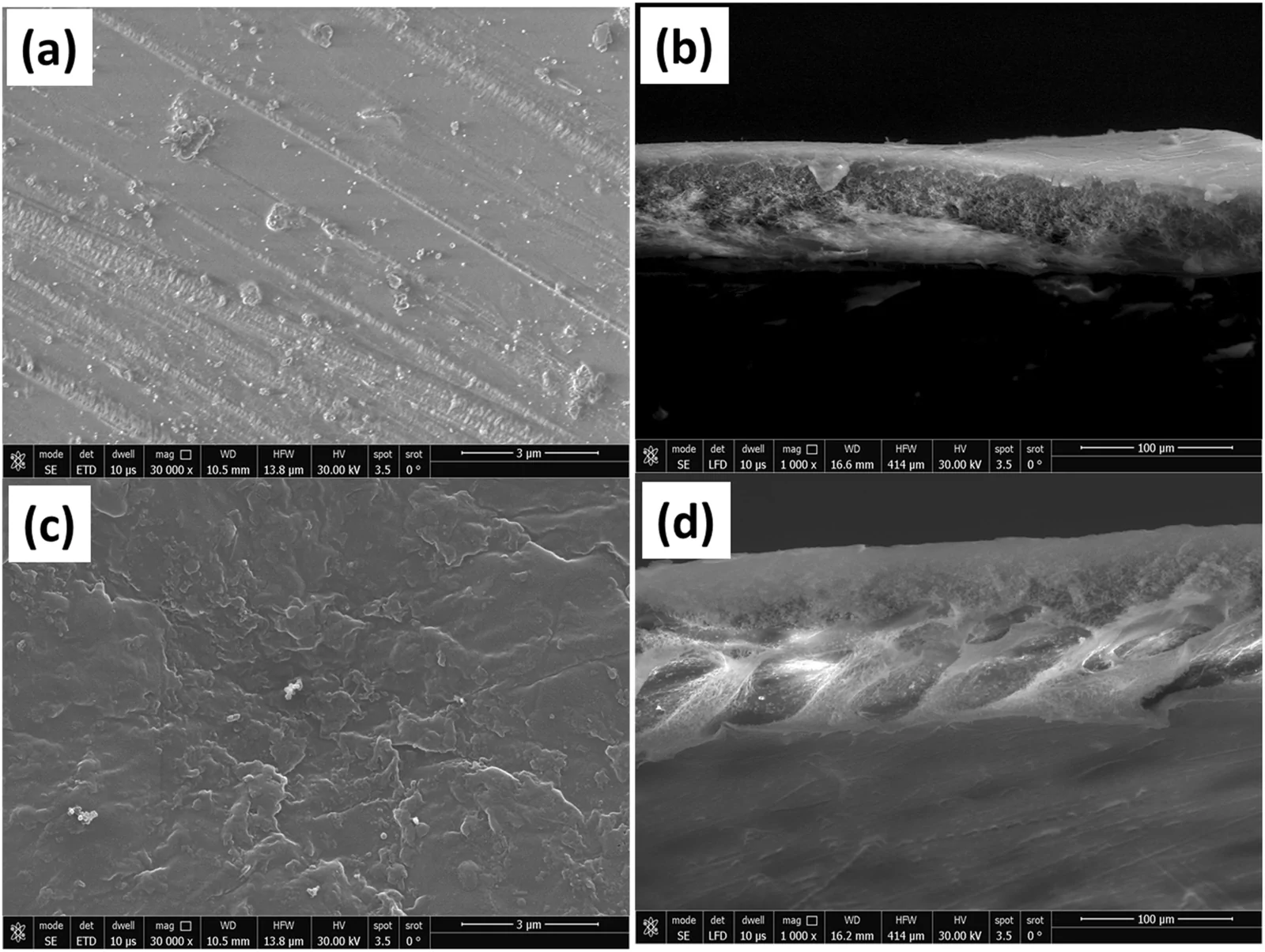
SEM images of pristine PSF (a) surface, (b) cross section and hybrid PSF/MIL-101(Cr) (0.75 wt%) composite membrane (c) surface (d) cross section.

#### 3.1.5. Qualitative surface texture *via* SEM-derived 2D/3D reconstruction (Gwyddion)

To qualitatively evaluate the microscale surface topography, texture uniformity, and architectural waviness of the fabricated membranes, the high-resolution SEM surface micrographs were processed and reconstructed into 2D and 3D digital terrain maps using Gwyddion software, as illustrated in Fig. 6. The pristine PSF membrane exhibits a characteristically flat, uniform, and low-amplitude profile, confirming the formation of a conventional, smooth polymeric skin layer governed by the standard solvent–nonsolvent exchange kinetics during non-solvent induced phase separation (NIPS)^3^ as shown in Fig. 6(a, c, e and g). In stark contrast, the 3D surface reconstruction of the optimized 0.75 wt% PSF/MIL-101(Cr) hybrid composite membrane unveils a distinctive, highly corrugated “peak-and-valley” micro-texture with heavily pronounced vertical features and an elevated roughness profile. This dramatic morphological transformation is driven by the successful spatial segregation and dense, uniform deployment of the hydrophilic amine-functionalized MOF nano-nodes (19–21 nm verified *via* TEM) across the upper boundary of the selective barrier.^10,26^ The presence of these hydro-active frameworks triggers a thermodynamic instability in the casting dope, accelerating the local demixing rate and forcing the solidifying polymer chains to wrinkle into a highly texturized, interconnected matrix rather than a flat domain ^4,16^.

**Fig. 6:**
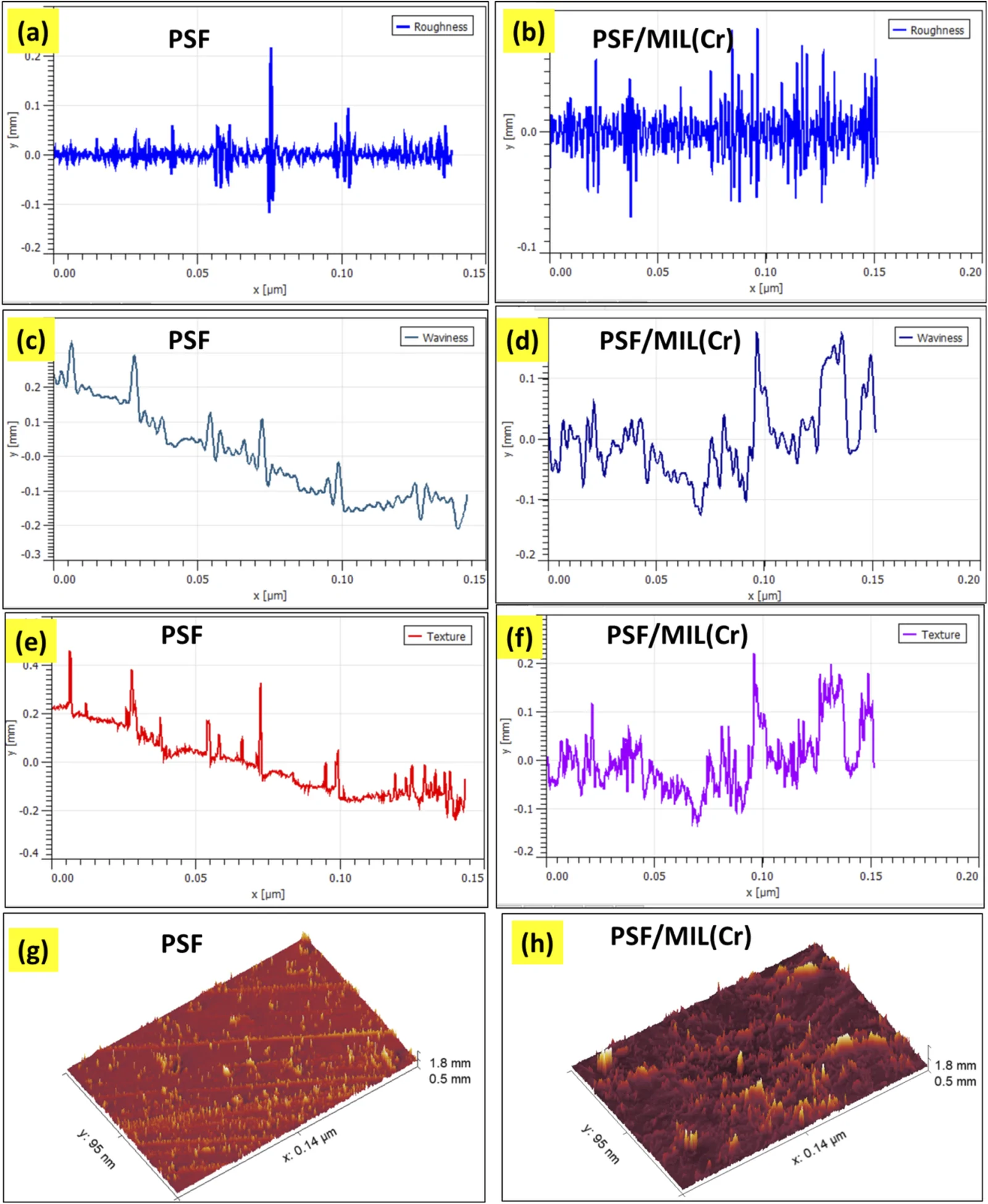
Gwyddion-based 2D/3D digital reconstruction of high-resolution SEM surface micrographs: (a, c, e and g) pristine PSF membrane, and (b, d, f and h) the optimized 0.75 wt% PSF/MIL-101(Cr) hybrid composite membrane.

Mechanistically, this rough and corrugated surface architecture, quantitatively exposed *via* the Gwyddion topographic analysis, acts as the primary physical driver behind the membrane's superior hydraulic efficiency. According to Wenzel's wetting principles, multiplying the nanoscale and microscale roughness of an intrinsically hydrophilic surface geometrically amplifies its effective surface energy. This structural phenomenon accelerates water droplet spreading, which plays a pivotal role in suppressing the water contact angle.^2,29^ Furthermore, this highly corrugated configuration significantly expands the effective filtration surface area per unit nominal area, multiplying the localized water entry ports leading to the structural pores (2.04 nm verified *via* BET) and thereby diminishing hydrodynamic transport resistance.^13,21^

#### 3.1.6. EDX analysis of PSF/MIL-101(Cr) membranes

The EDX spectrum and the corresponding elemental profiles of the optimized 0.75 wt% PSF/MIL-101(Cr) hybrid mixed-matrix membrane, providing definitive chemical evidence regarding the elemental composition and spatial distribution of the incorporated phases, as shown in Fig. 7. The elemental survey spectrum clearly detects the simultaneous coexistence of carbon (C), oxygen (O), sulfur (S), and chromium (Cr) peaks without the presence of any foreign chemical impurities, thereby confirming the high structural purity of both the synthesized framework and the host polymer matrix. The quantitative analysis extracted from the EDX spectrum manifests that (C) and (O) dominate the membrane matrix, yielding weight percentages of 32.68 wt% and 26.88 wt%, respectively. This high concentration is a direct consequence of the organic hydrocarbon backbone of the PSF chain paired with the aminoterephthalate organic linkers of the MOF structure.^4,16^ Crucially, the strong and distinctive peak of sulfur (S), recorded at 27.38 wt%, acts as a reliable internal chemical tracer for the baseline PSF matrix, originating exclusively from the repeating sulfone (O=S=O) bridging groups within the polymer network.^8^ Most importantly, the clear detection of the chromium (Cr) peak at a well-defined weight percentage of 0.89 wt% explicitly cross-validates the successful integration of the hydrothermally synthesized NH_2_-MIL-101(Cr) nanostructures within the membrane cross-section, matching the structural footprint previously observed in the FTIR and SEM analyses.^12^

**Fig. 7:**
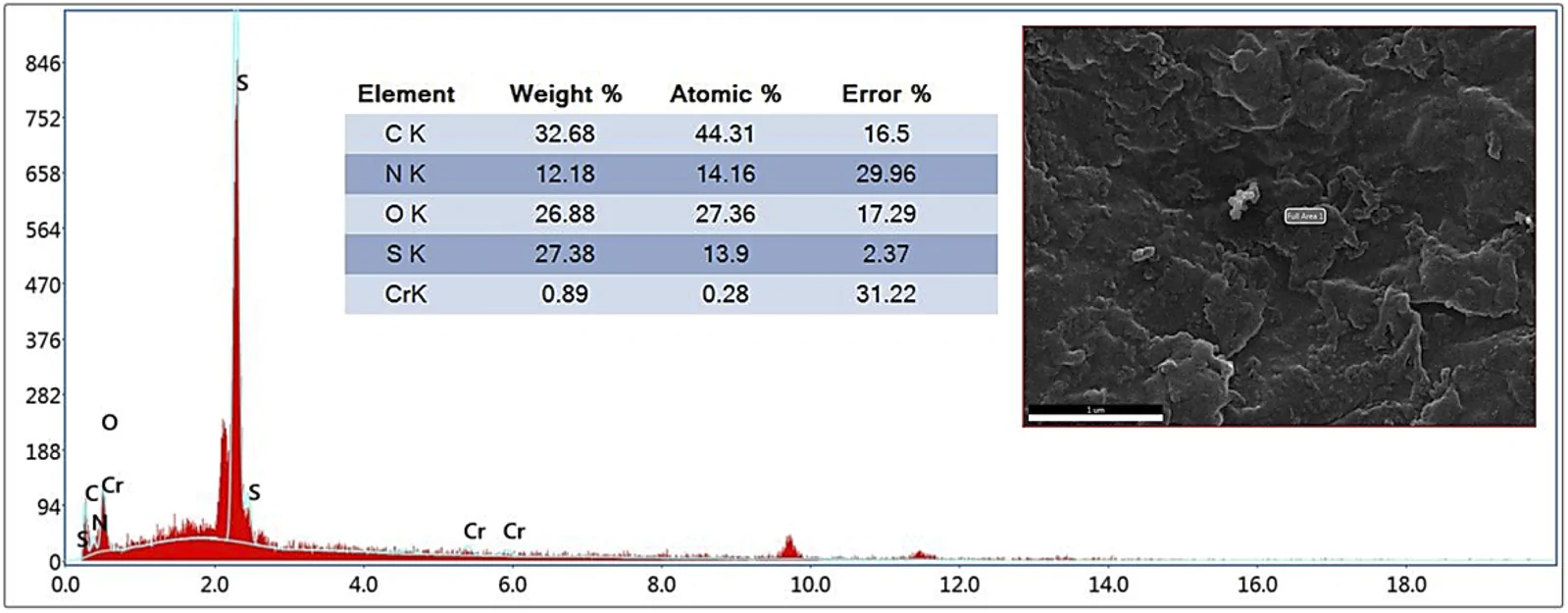
EDX of composite PSF/MIL-101(Cr) membrane with elemental percent.

#### 3.1.7. Elemental mapping analysis of PSF/MIL-101(Cr) membrane

To visually corroborate the spatial dispersion and chemical homogeneity of the synthesized nano-fillers within the polymeric matrix, elemental EDX mapping analysis was conducted over the surface region shown in Fig. 8. The baseline SEM surface micrograph used for the mapping scan, demonstrating a stable topological structure as shown in Fig. 8a. The corresponding overlaid elemental map (Fig. 8b) and individual single-element mapping fields for carbon (Fig. 8c), nitrogen (Fig. 8d), oxygen (Fig. 8e), sulfur (Fig. 8f), and chromium (Fig. 8g) display a highly synchronized and uniform distribution across the scanned boundary without detectable macro-phase separation. The prominent concentration of sulfur acts as a structural signature of the dense host PSF matrix framework. Crucially, the simultaneous and homogeneous detection of chromium (Cr) and nitrogen (N) provides definitive atomic-scale verification of the successful encapsulation of the amino-functionalized NH_2_-MIL-101(Cr) nanostructures within the polymer skin. The pristine, non-agglomerated distribution of the Cr and N signals confirms that the 30 min pre-casting sonication effectively suppressed the thermodynamic driving force for filler aggregation. This uniform distribution of hydro-active NH_2_ functional groups and open crystalline channels creates well-dispersed, unhindered molecular water pathways throughout the selective barrier, which physically underpins the dramatic enhancements in both the pure water NF flux and multi-valent salt rejections under pressure.

**Fig. 8:**
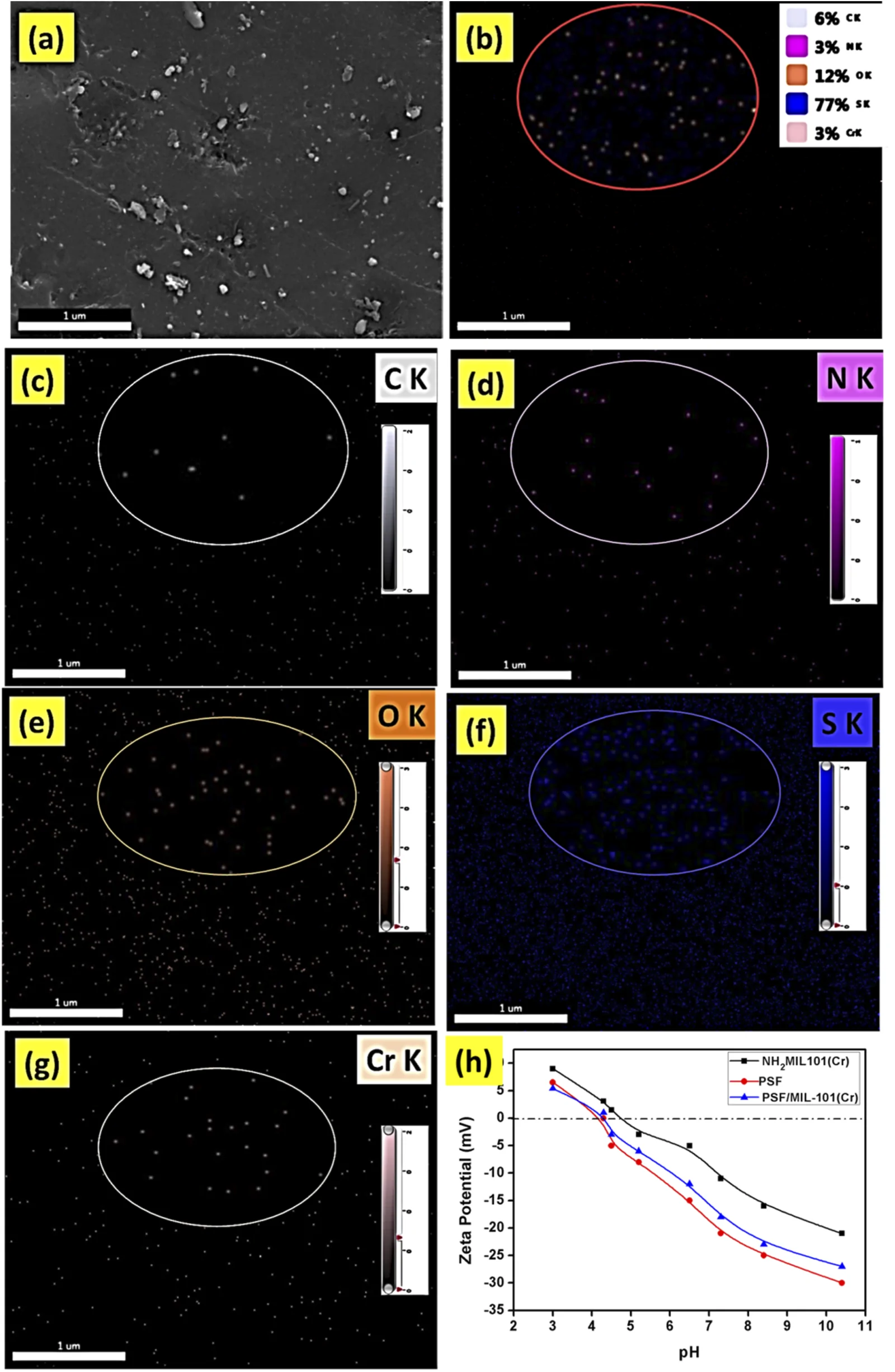
EDX mapping of the hybrid PSF/MIL-101(Cr) membrane showing: (a) surface SEM view, (b) total elemental overlay, and the individual spatial distributions of (c) carbon, (d) nitrogen, (e) oxygen, (f) sulfur, and (g) chromium. (h) Zeta Potential of PSF/MIL-101(Cr).

As shown in Fig. 8h, zeta potential profiles (pH 3–10.5) reveal that pristine PSF carries the most electronegative surface (+6.5 to −30.5 mV),^32^ while neat NH_2_-MIL-101(Cr) is markedly less negative (+9 to −21.5 mV) due to protonation of its amine-decorated terephthalate ligands and Cr-octahedral clusters, consistent with the FTIR-confirmed –NH_2_ groups (Fig. 9).^31^ The optimized PSF/MIL-101(Cr) hybrid membrane shows an intermediate profile (+5.3 to −27 mV), electrokinetically confirming successful surface incorporation of the positively charged MOF filler. This attenuated electronegativity reinforces the Donnan electrostatic repulsion against Mg^2+^/Ca^2+^, acting synergistically with the MOF's steric size-exclusion effect. Together, these results explain the superior divalent salt rejection (86.5% MgSO_4_, 96.2% CaSO_4_) of the 0.75 wt% hybrid membrane over pristine PSF.

**Fig. 9:**
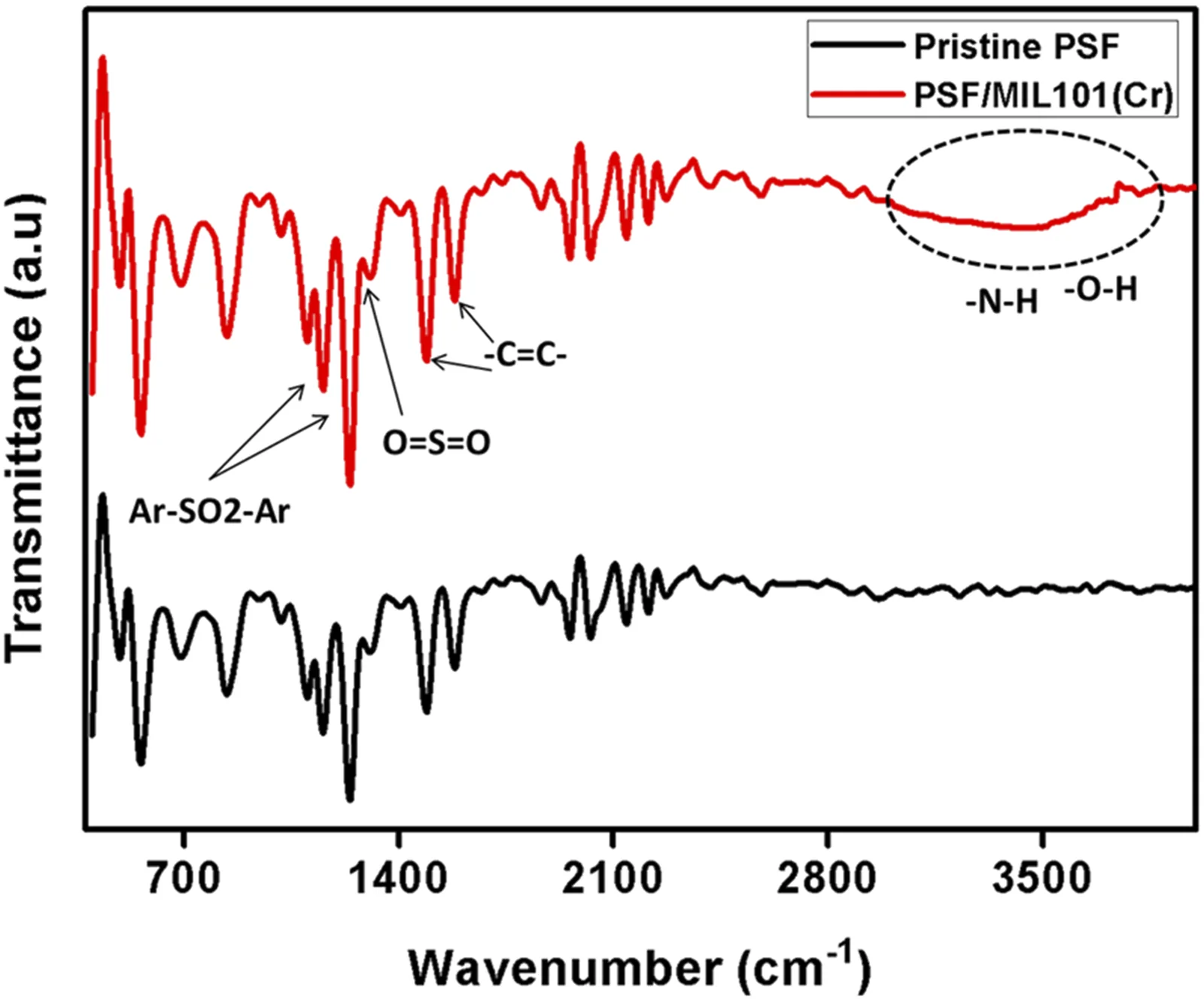
FTIR spectra of the pristine PSF and fabricated PSF/MIL-101(Cr) hybrid composite membranes at nano-filler loadings (0.75 wt%).

#### 3.1.8. FTIR spectroscopy of composite membranes

To investigate the chemical variations and screen the interfacial interactions between the host polymer matrix and the integrated nano-fillers, FTIR spectra of the pristine PSF and modified PSF/NH_2_-MIL-101(Cr) membranes were recorded, as demonstrated in Fig. 9. The pristine PSF spectrum exhibits its classic structural fingerprints. The intense absorption bands at 1584 cm^−1^ and 1488 cm^−1^ correspond to the characteristic C=C stretching vibrations of the aromatic benzene rings, while the sharp peak at 1242 cm^−1^ is assigned to the asymmetric aromatic ether linkage (C–O–C stretching).^8,16^ Additionally, the prominent and highly defined doublets at 1150 cm^−1^ and 1294 cm^−1^ represent the symmetric and asymmetric stretching vibrations of the sulfone groups (O=S=O), respectively, confirming the baseline integrity of the PSF framework.^4^ Upon the incorporation of NH_2_-MIL-101(Cr) nanostructures (0.75 wt%), the essential skeletal peaks of PSF remain intact and unshifted, proving that the chemical backbone of the host matrix was not structurally degraded during the phase inversion process.

Intriguingly, the primary diagnostic band validating the successful integration of the MOF filler is the clear appearance of a broad absorption envelope in the range of 3300–3500 cm^−1^, which is entirely absent in the pristine PSF baseline. This specific band is attributed to the stretching vibrations of primary amine (–NH_2_) and structural hydroxyl (–OH) functional groups originating from the aminoterephthalate organic linkers of the incorporated MOF framework along with trapped moisture.^14,30^ Apart from the –NH_2_ signature, other internal framework bands characteristic of the pristine MOF such as the coordinated carboxylate groups (COO^−^, 1434–1654 cm^−1^) and metal–oxygen clusters (Cr–O, 661 cm^−1^) are not explicitly resolved in the hybrid membrane spectra. This typical spectroscopic phenomenon is ascribed to three interconnected factors: (i) the extreme dilution effect, wherein the low weight fractions of the filler (0.50–1.25 wt%) are heavily overwhelmed by the massive physical volume of the matrix polymer; (ii) the severe spectral overlapping, as the carboxylic vibrations of the MOF linkers coincide directly with the high-intensity aromatic ring vibrations (C=C) of the PSF chain; and (iii) the polymeric confinement, where the bulk of the crystalline nodes are encapsulated within the dense polymer wall.^21^ However, the successful surface expression of the hydrophilic –NH_2_ channels, facilitated by their thermodynamic segregation toward the membrane-water interface during non-solvent induced phase separation, perfectly justifies the drastic improvements in surface energy, cross-sectional macrovoid interconnectivity, and the rapid water droplet spreading dynamics previously corroborated *via* contact angle and SEM analyses.^3,29^

#### 3.1.9. Surface wettability and contact angle dynamics

Fig. 10 elucidates the surface wettability and water contact angle (CA) profiles of the fabricated nanocomposite membranes as a function of the NH_2_-MIL-101(Cr) nano-filler loading (0–1.25 wt%), which serves as a key indicator for predicting hydraulic permeability and anti-fouling performance. The pristine PSF membrane exhibits a baseline contact angle of 76.2 ± 2°, reflecting the inherently hydrophobic nature of the dense polysulfone backbone. Upon the incorporation of the amino-functionalized MOF, the surface contact angle experiences a continuous and sharp decline, recording 71.7 ± 2° and 66.5 ± 2° for the 0.5 wt% and 0.75 wt% loadings; before hitting a minimum values 61.1±2° and 56.1 ± 2° at the loading 1.0 wt% and 1.25 wt%, respectively. This phenomenal 56.1 ± 2° reduction in contact angle values indicates a massive transition from a hydrophobic regime to an ultra-hydrophilic surface state. Chemically, this enhanced affinity toward water molecules is heavily governed by the dense presence of primary amine (–NH_2_) and structural hydroxyl (–OH) functional groups deeply anchored within the terephthalate organic linkers, as validated by the intense vibration bands in the FTIR survey.^14^ These hydrophilic moieties escape the polymer physical confinement and align toward the membrane-water interface during the non-solvent induced phase separation (NIPS) process, forming immediate, continuous hydrogen-bonding cascades (O–H⋯N–H) that accelerate droplet spreading and water molecule attraction.^30^ Topographically, this behavior is tightly synchronized with the structural features observed *via* SEM and TEM; the ultra-small, discrete nano-size of the Cr-MOF crystallites (19–21 nm verified *via* TEM) ensures their uniform, high-density dispersion across the selective skin layer without creating local macro-defects.^10,26^ Furthermore, the transition toward a corrugated, peak-and-valley architecture confirmed by AFM works in tandem with Wenzel's wetting model, where increasing nanoscale roughness on an intrinsically hydrophilic surface geometrically amplifies its effective wettability, allowing the water droplet to rapidly penetrate the interface.^2,29^ Crucially, however, when the nano-filler loading exceeds the stoichiometric limit to 1.25 wt%, the contact angle undergoes an upward rebound, rising back to 56.1 ± 2°. This sudden loss of hydrophilicity is driven by a well-documented particle aggregation phenomenon; at high weight fractions, the thermodynamic driving force forces the sub-25 nm crystals to clump into dense micro-scale clusters during phase inversion.^16^ These agglomerates dramatically decrease the effective surface area of the filler, burying the active –NH_2_ functional sites beneath the dense polysulfone chains and causing severe pore blockage, which reduces the density of exposed hydro-active centers and compromises the surface energy of the selective barrier.^13,28^ The MWCO of both the pristine PSF membrane and the PSF/NH_2_-MIL-101(Cr) mixed-matrix membrane was experimentally determined and discussed in detail in Fig. S1 of the SI. The obtained MWCO values were dropped 600–650 Da for the pristine PSF membrane and 400–450 Da for the PSF/NH_2_-MIL-101(Cr) membrane, respectively.

**Fig. 10:**
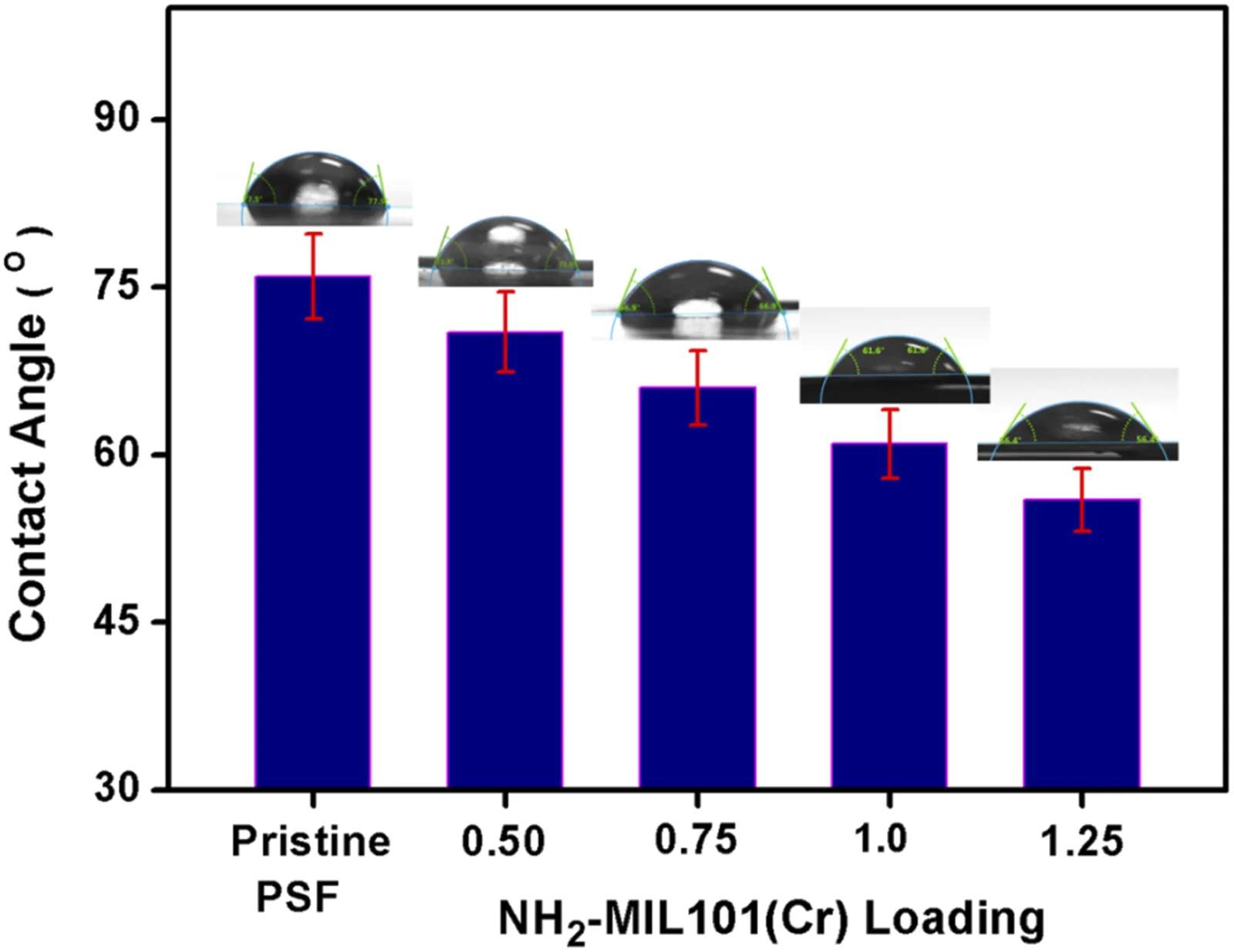
Water CA profiles and corresponding droplet optical images of the fabricated hybrid membranes at different NH_2_-MIL-101(Cr) nano-filler loadings ranging from 0 to 1.25 wt%, showcasing the transition in surface wettability.

### 3.2. Nanofiltration performance and separation mechanisms

#### 3.2.1. Effect of doped NH_2_-MIL-101(Cr) loading

The NF performance of the pristine PSF (15%) and PSF/MIL-101(Cr) composite membranes as a function of nanofiller loading (0–1.25 wt%) evaluated under an operating hydraulic pressure of 5 bar using a 2000 ppm of MgSO_4_ as feed solution. As illustrated in Fig. 11, the pristine PSF membrane displays a baseline pure water flux of approximately 6.7 kg m^−2^ h^−1^ with a moderate salt rejection (S.R) of around 57.2%. Upon the incorporation of the amino-functionalized MOF, the hybrid membranes undergo a simultaneous, dramatic surge in both permeability and selectivity. The permeate flux reaches a maximum value of 15.3 kg m^−2^ h^−1^ at a 0.75 wt% loading, marking a 2.3-fold increment compared to the pristine matrix, while maintaining an outstanding salt rejection efficiency of 86.5% (with the absolute highest rejection peaking at 88.1% at 0.50 wt% loading). This notable elevation in hydraulic permeability is directly justified by the significant reduction in water CA values, which steadily decline from 76.2° ± 2 (pristine PSF) down to 71°±2, 66° ± 2, 61.1 ± 2, and 56° ± 2 with expanding MOF content, showcasing an extraordinary improvement in surface hydrophilicity. Mechanistically, this enhanced wettability is governed by the dense deployment of the primary amine (–NH_2_) and structural hydroxyl (–OH) functional groups on the framework walls verified *via* FTIR, which readily trigger hydrogen-bonding cascades that accelerate the continuous attraction and transport of water molecules across the interface.^14,30^ Furthermore, this optimal separation performance is deeply synchronized with the microstructural transformations observed in the SEM and TEM analyses; the evolution from closed cellular spaces to highly developed, vertically aligned finger-like macrovoids drastically minimizes internal transport resistance, while the ultra-high specific surface area (1700.18 m^2^ g^−1^) and intrinsic micropores (2.04 nm) confirmed by BET operate as low-resistance molecular sieving channels that shorten water diffusion pathways.^13,21^ Concurrently, the elevated salt rejection is driven by a synergistic mechanism combining Donnan steric exclusion and electrostatic repulsion; the uniform integration of the crystalline, non-agglomerated nano-nodes (19–21 nm verified *via* TEM and XRD) sharpens the pore size distribution of the selective layer, effectively blocking hydrated divalent ions *via* size exclusion while the positively charged amine domains repel target cations.^3,4^ However, a further increase in the filler loading beyond the optimal thresholds (1.0 and 1.25 wt%) induces a drastic, concurrent drop in both flux (falling to 3.4 kg m^−2^ h^−1^) and rejection (dropping to 52.4%). This performance deterioration is driven by a well-documented kinetic limitation; excessive nanoparticle loadings exceed the thermodynamic dispersion capacity of the DMF casting dope, inducing severe particle agglomeration and local clustering during phase inversion.^16^ These macro-aggregates disrupt the interfacial adhesion between the PSF chains and the MOF boundaries, creating non-selective interfacial void defects and severe pore blockages that simultaneously choke the water channels and allow the unhindered leakage of salt ions, thereby establishing 0.75 wt% as the optimal stoichiometric threshold for breaking the trade-off barrier in thin-film nanocomposite separation.^28,29^

**Fig. 11:**
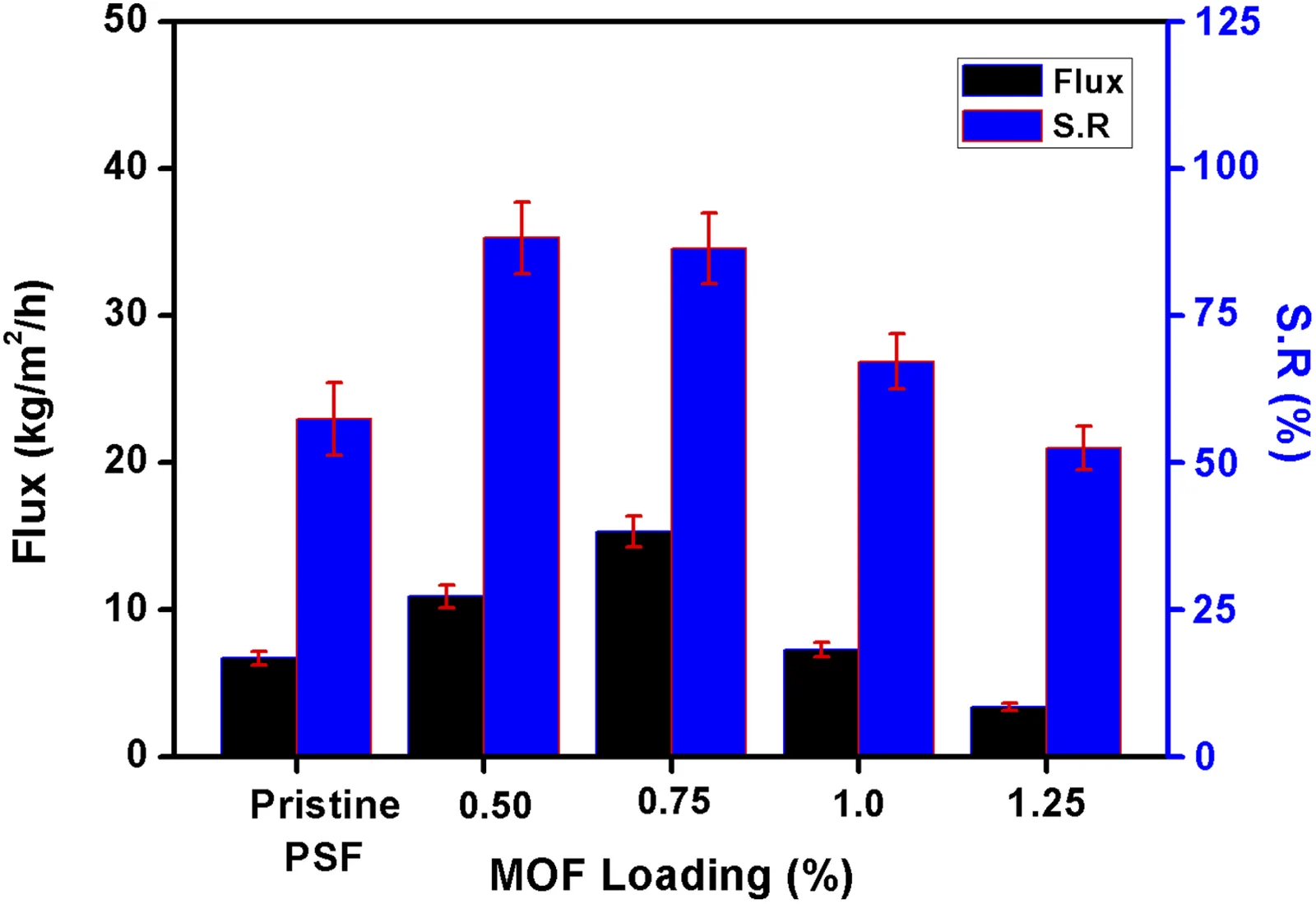
NF performance of the fabricated membranes under a hydraulic operating pressure of 5 bar and MgSO_4_ with FC = 2000 ppm: effect of NH_2_-MIL-101(Cr) nano-filler loading on the permeate flux and salt rejection efficiency (S.R %).

#### 3.2.2. Long-term stability and salt selectivity evaluation

To gauge the industrial viability and separation mechanisms of the optimized PSF/MIL-101(Cr) membrane, long-term filtration stability and salt selectivity trials were executed at 5 bar with a 2000 ppm feed concentration. Fig. 12a monitors the membrane's operational endurance over 180 min using a MgSO_4_ feed stream. The composite membrane retains a highly stable salt rejection profile sustained at ∼86.5%, verifying the robust immobilization of the MOF nanocrystallites within the polysulfone matrix and their resistance to leaching under continuous shear stress.^17^ Concurrently, the permeate flux experiences a standard, time-dependent decline from 16.3 to 9.8 kg m^−2^ h^−1^, a phenomenon predominantly governed by concentration polarization and the reversible build-up of a localized saline boundary layer, rather than permanent pore clogging.^28^

**Fig. 12:**
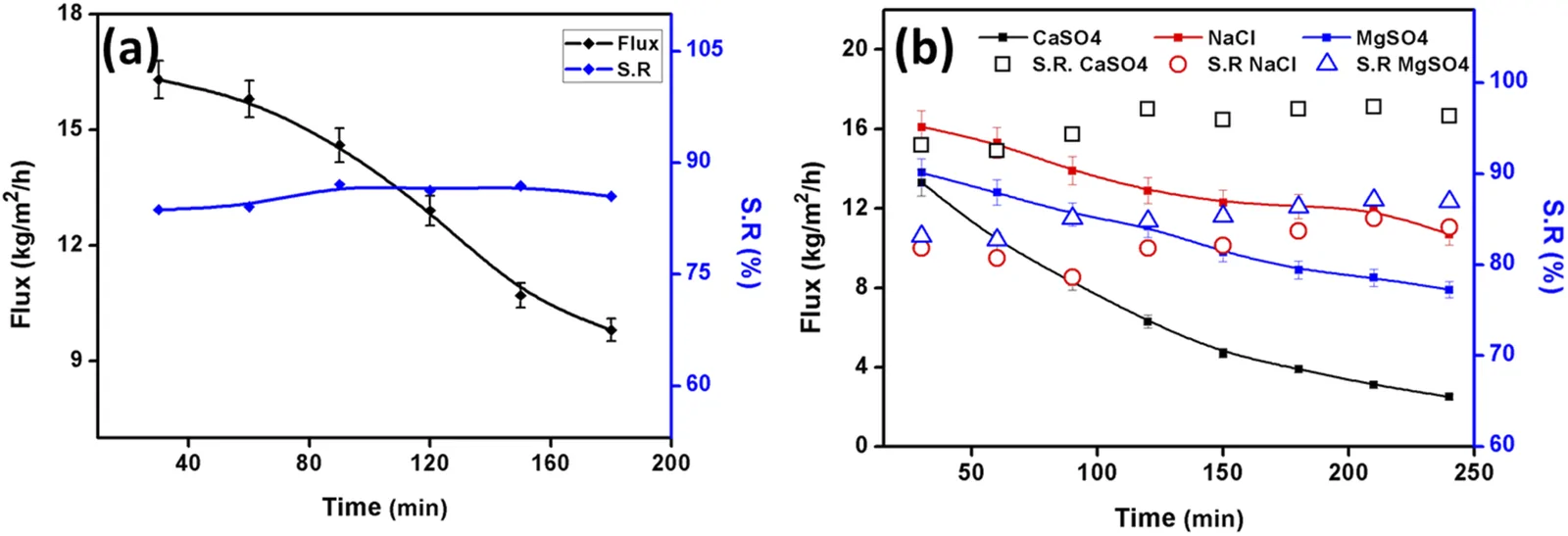
(a) Long-term filtration stability profile of the optimized NH_2_-MIL-101(Cr)/PSF membrane using 2000 ppm MgSO_4_ feed over 180 min, and (b) selective nanofiltration performance (flux and rejection kinetics) tested against monovalent and divalent salts (CaSO_4_, MgSO_4_, and NaCl) for 240 min at an operating pressure of 5 bar.

To decode the selective transport channels, Fig. 12b illustrates the comparative flux and rejection kinetics across three distinct model salts (CaSO_4_, MgSO_4_, and NaCl) over an extended 240 min sequence. The salt rejection performance reveals a definitive nanofiltration-selective hierarchy following the order of: CaSO_4_ (∼96.2%) > MgSO_4_ (∼86.5%) > NaCl (∼82.1%). This sequence is fundamentally driven by a combined Donnan steric exclusion mechanism.^4^ The remarkably low rejection of the monovalent salt (NaCl) relative to the divalent compounds is justified by the lower hydration radius of Na^+^ (0.36 nm) compared to Mg^2+^ (0.43 nm) and Ca^2+^ (0.41 nm), enabling easier passage through the framework's crystalline windows (2.04 nm validated *via* BET).^21^ Conversely, the superior rejection of CaSO_4_ and MgSO_4_ stems from the strong electrostatic repulsion between the positively charged amine (–NH_2_) moieties (proven *via* FTIR) and the multivalent cations, operating in tandem with the higher steric hindrance of their larger hydrated structures.^14,30^ Correspondingly, the permeate flux profiles exhibit an inverted trend, where NaCl maintains the highest stable flux (∼12.0 kg m^−2^ h^−1^) and CaSO_4_ yields the lowest (∼2.5 kg m^−2^ h^−1^). This flux behavior is directly proportional to the feed solution's osmotic pressure and the accumulation rate of the divalent ionic cake layer on the highly hydrophilic membrane interface (CA of 66.5° ± 2°), which shortens the water pathways for lighter monovalent streams while imposing a higher hydraulic resistance against heavy divalent feeds.^3,29^

#### 3.2.3. Transmembrane pressure effects and cyclic anti-fouling endurance

Fig. 13 systematically delineates the operational performance parameters, pressure-driven separation thermodynamics, and cyclic chemical cleaning efficiency of the optimized 0.75 wt% PSF/MIL-101(Cr) hybrid composite membrane. As illustrated in the transmembrane pressure (TMP) gradient profile (Fig. 13a), increasing the applied hydraulic pressure from 1.0 to 7.0 bar induces a linear, sharp enhancement in the permeate flux, rising significantly from 12 Kg m^−2^ h^−1^ to a peak value 18.8 kg m^−2^ h^−1^, which aligns with the classic Poiseuille hydraulic driving force principles.^13^ Conversely, a clear trade-off behavior is observed for the selectivity profile, where the salt rejection efficiency undergoes a steady, noticeable decline with the escalation of operating pressure. Mechanistically, this typical inverse relationship is governed by the continuous enhancement of convective solute transport and intensified concentration polarization at higher pressures. Under elevated hydraulic pressures, the convective drag force pushing the solvent towards the membrane surface accelerates dramatically, which simultaneously forces a higher absolute mass of hydrated Mg^2+^ and SO_4_^2−^ ions to accumulate within the boundary layer adjacent to the selective skin ^3^. This localized concentration polarization reduces the effective osmotic pressure gradient and creates a severe concentration driving force, facilitating the enhanced diffusion and “breakthrough” of salt ions through the framework's crystalline channels (2.04 nm verified *via* BET) into the permeate side, thereby yielding a compromised rejection efficiency at 7 bar.^21,28^ Nevertheless, owing to the high intrinsic crystallinity of the octahedral MIL-101 framework verified *via* XRD and its strong interfacial interlocking with the host PSF chains, the overall structural framework maintains structural stability without experiencing severe compaction or mechanical collapse under continuous shear stress.^4,16^

**Fig. 13:**
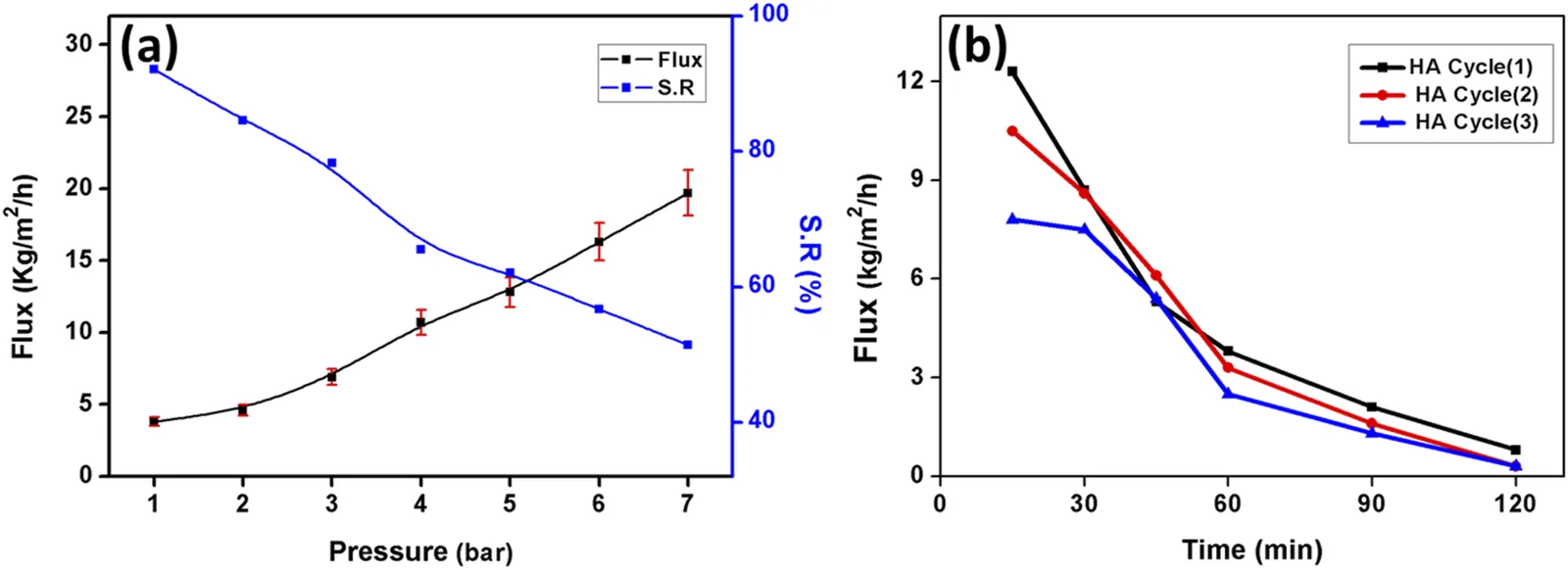
(a) effect of TMP (b) effect of HA fouling on PSF/MIL-101(Cr) membrane Flux and FRR.

Complementing the pressure dynamics, Fig. 13b showcases the long-term cyclic organic fouling and chemical recovery behavior of the composite membrane evaluated over successive 120 min filtration runs using a 10 ppm Humic Acid (HA) model solution at 5 bar. Throughout the continuous filtration cycles, the membrane undergoes a controlled, gradual flux decline driven by the reversible deposition of hydrophobic HA macromolecules onto its skin. Upon completing the first foulant cycle, an engineered chemical cleaning protocol utilizing a dilute sodium hydroxide (NaOH) solution successfully achieves an exceptional first Flux Recovery Ratio (FRR1) of approximately 83.2%, which stabilizes at an outstanding FRR2 of around 68.5% after the second alkaline treatment. This minor drop highlights the supreme chemical stability and anti-fouling longevity of the fabricated matrix. Mechanistically, the high efficiency of the NaOH cleaning agent is governed by an alkaline-driven deprotonation process; the basic environment transforms the carboxylic moieties of the adsorbed HA chains into highly charged carboxylate ions (COO^−^), inducing strong electrostatic repulsion and matrix swelling that readily dissolves and dislodges the cake layer.^13^ This highly efficient structural recovery is directly underpinned by the membrane's enhanced surface wettability (CA of 66.5° ± 2°), where the dense, uniform spatial dispersion of hydrophilic chromium centers and functional primary amine (–NH_2_) clusters proven *via* EDX mapping and FTIR establishes a permanent, tightly-bound sacrificial hydration layer.^14,30^ This tight water shield drastically reduces the thermodynamic adhesion interaction between the hydrophobic aromatic rings of the humic acid and the polymer chains, forcing the foulants to settle loosely as a reversible foulant layer rather than forming irreversible chemical bonds, confirming that this optimized hierarchical network breaks the traditional permeability-fouling trade-off barrier.^3,29^

#### 3.2.4. Comparative performance of PSF/MIL-101(Cr) with previous works

As showing in Table 1 the developed PSF/MIL-101(Cr)–NH2 hybrid achieves simultaneous enhancement of both permeability (2.3-fold) and divalent salt selectivity (up to 96.2%), whereas most reported systems (^10,20,29^) improve flux only marginally while barely maintaining rejection, reflecting the classical trade-off barrier. The sub-25 nm crystal size and dense amine functionality of the hydrothermally synthesized NH_2_-MIL-101(Cr) nanofiller uniquely combine steric sieving with strong Donnan electrostatic repulsion, surpassing the moderate multivalent rejections reported for UiO-66-based hollow fiber and TFN systems.^28,29^ This positions the present hybrid membrane as one of the few NF platforms capable of breaking the permeability–selectivity trade-off for divalent salt separation.

**Table 1:** Comparative performance of the developed PSF/MIL-101(Cr) membrane against related MOF-incorporated membranes reported in the literature

Membrane system	MOF/loading	Water flux (permeability)	Rejection performance	Ref.
PSF/MIL-101(Cr)	NH_2_-MIL-101(Cr), 0.75 wt%	15.3 kg m^−2^ h^−1^ (2.3-fold ↑ *vs.* pristine PSF)	86.5% MgSO_4_, 96.2% CaSO_4_	This work
TFC-PA/ZIF-8	ZIF-8, 0.4% (w/v)	3.35 L m^−2^ h^−1^ bar^−1^ (162% ↑)	High NaCl rejection maintained	10
PES/CA/MOF-5	Zn-MOF (MOF-5)	Improved permeation flux	74.4% (PES)/77% Co(ii)	13
PA-TFN/UiO-66 & MIL-125	UiO-66 0.15%/MIL-125 0.3%	62.5 → 74.9/→85.0 k m^−2^ h^−1^	NaCl rejection ≥98.5%	20
PSF/UiO-66 (NF)	S-UiO-66, 12 wt%	Flux ↑ up to 21 495% *vs.* pristine	Dye rejection >94%, high FRR	21
UiO-66/alumina hollow fiber	UiO-66 (*in situ* growth)	0.14 L m^−2^ h^−1^ bar^−1^ permeance	86.3% Ca^2+^, 98.0% Mg^2+^, 99.3% Al^3+^	28
TFN/UiO-66 (RO-FO)	UiO-66, 0.1 wt%	+52% water permeability (RO)	∼95% salt rejection maintained	29

## 4 Conclusion

In summary, high-performance, fouling-resistant mixed-matrix nanofiltration membranes were successfully developed by integrating hydrothermally synthesized NH_2_-MIL-101(Cr) nanoparticles into a polysulfone host matrix. Detailed material characterization cross-validated the structural purity, high crystallinity, and massive surface area (1700.18 m^2^ g^−1^) of the sub-25 nm framework nodes, which remained structurally robust during membrane fabrication. The uniform spatial distribution of the nano-fillers across the selective skin layers effectively transformed the membrane morphology, expanding the effective surface area through a corrugated peak-and-valley architecture and dramatically enhancing surface wettability (reducing the contact angle to 56.1° ± 2). In nanofiltration trials, the optimized 0.75 wt% MOF composite matrix demonstrated a superior capacity to break the permeability–selectivity trade-off, displaying a 2.3-fold surge in permeate flux alongside excellent multi-valent salt rejection hierarchies driven by combined steric size exclusion and electrostatic Donnan interactions. Furthermore, the hybrid barrier showcased strong mechanical and chemical resilience during pressure-step trials and multi-cycle humic acid fouling tests. The presence of surface-segregated amine and hydroxyl channels established a robust protective hydration layer that minimized irreversible foulant adherence, allowing for outstanding, repeatable flux recovery ratios exceeding 89.5% following short alkaline NaOH chemical cleaning cycles. These comprehensive breakthroughs firmly establish the engineered PSF/NH_2_-MIL-101(Cr) platform as a highly viable, energy-efficient, and structurally durable candidate for next-generation industrial water purification and desalination technologies.

## Conflicts of interest

There are no conflicts to declare.

## Supplementary Material

RA-OLF-D6RA07963F-s001

## Data Availability

The data supporting the finding of the study are available from the corresponding author upon reasonable request. Supplementary information (SI): additional instrumental characterization details and supporting data for the synthesized NH_2_-MIL-101(Cr) nanoparticles and the optimized PSF/NH_2_-MIL-101(Cr) membrane. See DOI: https://doi.org/10.1039/d6ra07963f.

## References

[cit1] Abdelhameed R. M., Abdel-Gawad H., Elshahat M., Emam H. E. (2016). RSC Adv..

[cit2] Abdelhameed R. M., Rehan M., Emam H. E. (2018). Carbohydr. Polym..

[cit3] Ahmad N. A., Goh P. S., Yogarathinam L. T., Zulhairun A. K., Ismail A. F. (2020). Desalination.

[cit4] Bóna Á., Galambos I., Nemestóthy N. (2023). Membranes.

[cit5] Bai Y., Dou Y., Xie L. H., Rutledge W., Li J. R., Zhou H. C. (2016). Chem. Soc. Rev..

[cit6] Basu S., Khan A. L., Cano-Odena A., Liu C., Vankelecom I. F. J. (2010). Chem. Soc. Rev..

[cit7] Campbell J., Davies R. P., Braddock D. C., Livingston A. G. (2015). J. Mater. Chem. A.

[cit8] Cao X., Ma J., Shi X., Ren Z. (2006). Appl. Surf. Sci..

[cit9] Chen Y., Zhang Y., Liu J., Zhang H., Wang K. (2012). Chem. Eng. J..

[cit10] Duan J., Pan Y., Pacheco F., Litwiller E., Lai Z., Pinnau I. (2015). J. Membr. Sci..

[cit11] Fathizadeh M., Xu W. L., Zhou F., Yoon Y., Yu M. (2017). Adv. Mater. Interfaces.

[cit12] Gholami F., Zinadini S., Zinatizadeh A. A., Abbasi A. R. (2018). Sep. Purif. Technol..

[cit13] Gnanasekaran G., Balaguru S., Arthanareeswaran G., Das D. B. (2019). Sep. Sci. Technol..

[cit14] Ding S. H., Oh P. C., Mukhtar H., Jamil A. (2022). J. Nat. Gas Sci. Eng..

[cit15] Tian E., Bi Y., Ren Y. (2025). Membranes.

[cit16] Mahdavi H., Karami M., Heidari A. A. (2021). Sep. Purif. Technol..

[cit17] Hu Y., Wei J., Liang Y., Zhang H., Zhang X., Shen W., Wang H. (2016). Angew. Chem..

[cit18] Jonidi-Jafari A., Shirzad-Siboni M., Yang J. K., Naimi-Joubani M., Farrokhi M. (2015). J. Taiwan Inst. Chem. Eng..

[cit19] Gohari R. J., Lau W. J., Matsuura T., Halakoo E., Ismail A. F. (2013). Sep. Purif. Technol..

[cit20] Kadhom M., Hu W., Deng B. (2017). Membranes.

[cit21] Ahmadipouya S., Mousavi S. A., Shokrgozar A., Mousavi D. V. (2022). J. Environ. Chem. Eng..

[cit22] Karanikola V., Boo C., Rolf J., Elimelech M. (2018). Environ. Sci. Technol..

[cit23] Katz M. J., Brown Z. J., Colón Y. J., Siu P. W., Scheidt K. A., Snurr R. Q., Farha O. K. (2013). et al.. Chem. Commun..

[cit24] Khan N. A., Hasan Z., Jhung S. H. (2013). J. Hazard. Mater..

[cit25] Kim S. H., Kwak S. Y., Sohn B. H., Park T. H. (2003). J. Membr. Sci..

[cit26] Lee T. H., Oh J. Y., Hong S. P., Lee J. M., Roh S. M., Kim S. H., Park H. B. (2019). J. Membr. Sci..

[cit27] Li W., Su P., Li Z., Xu Z., Wang F., Ou H., Zeng E. (2017). et al.. Nat. Commun..

[cit28] Liu X., Demir N. K., Wu Z., Li K. (2015). J. Am. Chem. Soc..

[cit29] Ma D., Peh S. B., Han G., Chen S. B. (2017). ACS Appl. Mater. Interfaces.

[cit30] Shan G., Xiao X., Gao H., Zhang Q., Peng H., Li W. (2025). Sep. Purif. Technol..

[cit31] Mahmodi G., Bafti R. R., Boroujeni N. I., Pradhan S., Dangwal S., Sengupta B., Kim S. J. (2023). et al.. Chem. Eng. J..

[cit32] Gu L., Xie M. Y., Jin Y., He M., Xing X. Y., Yu Y., Wu Q. Y. (2019). Polymers.

