# Supplementary material for: Polysulfone mixed matrix membranes embedded with sub-25 nm NH2-MIL-101(Cr) for enhanced divalent salt separation and permeability
Source: RSC Adv. 2026 Oct 14;16(47):62769–83. doi: 10.1039/d6ra07963f (PMC13662755; doi:10.1039/d6ra07963f)
Supplement: Supplementary file 1 [file RA-016-D6RA07963F-s001.pdf]

Supplementary Information (SI) for RSC Advances.
This journal is © The Royal Society of Chemistry

Polysulfone Mixed Matrix Membranes Embedded with Sub-25 nm NH_2-MIL-101(Cr) for Enhanced Divalent Salt Separation and Permeability.

Mubark Alshareef[1], Ahmed Alharbi[1], Mohamed S. Fahmi[2,*]

[1] *Department of Chemistry, Faculty of Science, Umm Al-Qura University, Makkah, Saudi Arabia;* *mmshreef@uqu.edu.sa* (M.A.)

[2] *Suez University, Faculty of Science, Chemistry Department, 43518 Suez, Egypt,* *masf23@sci.suezuni.edu.eg* (M.S.F.)

1. Characterization

The morphology and elemental composition of NH_2-MIL-101(Cr) and the membranes (surface and cross-section) were examined by SEM (QUANTA, FEG 250]) coupled with EDX and elemental mapping, and TEM (JEM-2100) for the nanoparticle size. Surface roughness and 3D topography were extracted from the SEM images using Gwyddion software (2.53). N_2 adsorption–desorption isotherms were recorded at 77 K (Quanta Chrome Autosorb-1 apparatus) after degassing at 150 ºC for 6 h, and the surface area and pore size were calculated by the BET and [BJH/NLDFT] methods, respectively. The phase formation of the samples was analyzed using X-ray diffraction (XRD) with Cu Kα radiation (λ = 0.154 nm). The scan range was set from 2θ = 10° to 80°, and the measurements were performed with a continuous scanning rate of 10°/min using a Bruker D8 Discover diffractometer, and the functional groups were identified by FTIR (ATR-FTIR SPECTROSCOPY, THERMO NICLOT, 50). The static water contact angle was measured by the sessile drop method (KRUSS, MODEL DSA)

at 25±2°C, and the reported values are averages of 5 measurements. The membrane surface charge was determined by zeta potential measurements (SurPASS electrokinetic). Salt concentrations in the feed and permeate were measured with a conductivity meter (Adwa, AD32), and PEG concentrations by TOC analyzer [Spectrophotometer].

2. Zeta Potential Measurement

The surface charge of the pristine PSF and the optimized PSF/NH₂-MIL-101(Cr) (0.75 wt%) membranes was evaluated by zeta potential measurements using a SurPASS electrokinetic analyzer, based on streaming potential and streaming current measurements. The membrane samples were mounted in an adjustable-gap clamping cell, and the measurements were performed in a KCl background electrolyte solution. The pH dependence of the zeta potential was obtained by titration over the pH range 3–10.5, using HCl (0.25 M) to lower the pH and NaOH, 0.1 M to raise it. The reported zeta potential values are the averages of measurements performed on two membrane samples of each type.

3. Pore Size and Molecular Weight Cut-Off (MWCO)

To clarify the size-sieving behavior of the selective layer, the rejection of neutral polyethylene glycol (PEG, 200–1200 Da) was measured for pristine PSF and the optimized 0.75 wt% PSF/MIL-101(Cr) membrane **(Figure S1)**. Both membranes show rejection increasing with PEG molecular weight, the typical signature of size exclusion. However, the hybrid membrane rejected more PEG across the entire range (≈69, 90.5, 93, 94.5 and 98% versus ≈61, 82, 89.5, 91.5 and 93% for pristine PSF at 200, 400, 600, 800 and 1200 Da). Taking the MWCO as the molecular weight at 90% rejection, it dropped from ≈600–650 Da for pristine PSF to ≈400–450 Da for the hybrid membrane. Using the Stokes-radius correlation of PEG:

$$dp = 33.46 \times 10^{-3} \times M^{0.557}$$ ....(3)

the effective pore radius decreased from ≈0.60 nm to ≈0.48 nm. The hybrid membrane is therefore tighter, even though its flux is 2.3-fold higher. This agrees with the improved rejection of hydrated Mg^{2}⁺ (0.43 nm) and Ca^{2}⁺ (0.41 nm) in Section 3.2.1, since the effective pore radius of the pristine membrane (≈0.60 nm) is well above the ion radii, while that of the hybrid (≈0.48 nm) approaches them [1]. The tighter skin layer is consistent with the uniform distribution of the 19–21 nm nanocrystals **(Figures 7–8),** which sharpen the pore size distribution of the skin. Meanwhile the finger-like macrovoids **(Figure 5d)** and the higher hydrophilicity **(Figure 10)** lower the resistance of the sublayer and the water entry, so flux and selectivity increase together. Note that the effective pore size (≈1.0 nm) is smaller than the average MOF pore (2.04 nm), so the sieving is governed mainly by the MOF-modified polymer skin rather than the MOF cavities alone. The higher $MgSO_4$ rejection (86.5%) compared with the neutral PEG of similar size (≈69% at 200 Da) also indicates an additional electrostatic (Donnan) contribution. This simultaneous gain in rejection and permeability agrees with previous reports on hydrophilic MOF-modified membranes: UiO-66 in thin-film nanocomposite membranes improved water flux and salt rejection together [2], UiO-66 in polysulfone NF membranes improved separation and antifouling [3], and MIL-53(Al) in polysulfone matrices altered the membrane structure and enhanced separation [4] . Similar to those works, the improvement here comes from better filler dispersion and hydrophilicity, and it disappears when the loading exceeds the optimum (≥1.0 wt%) because of agglomeration.

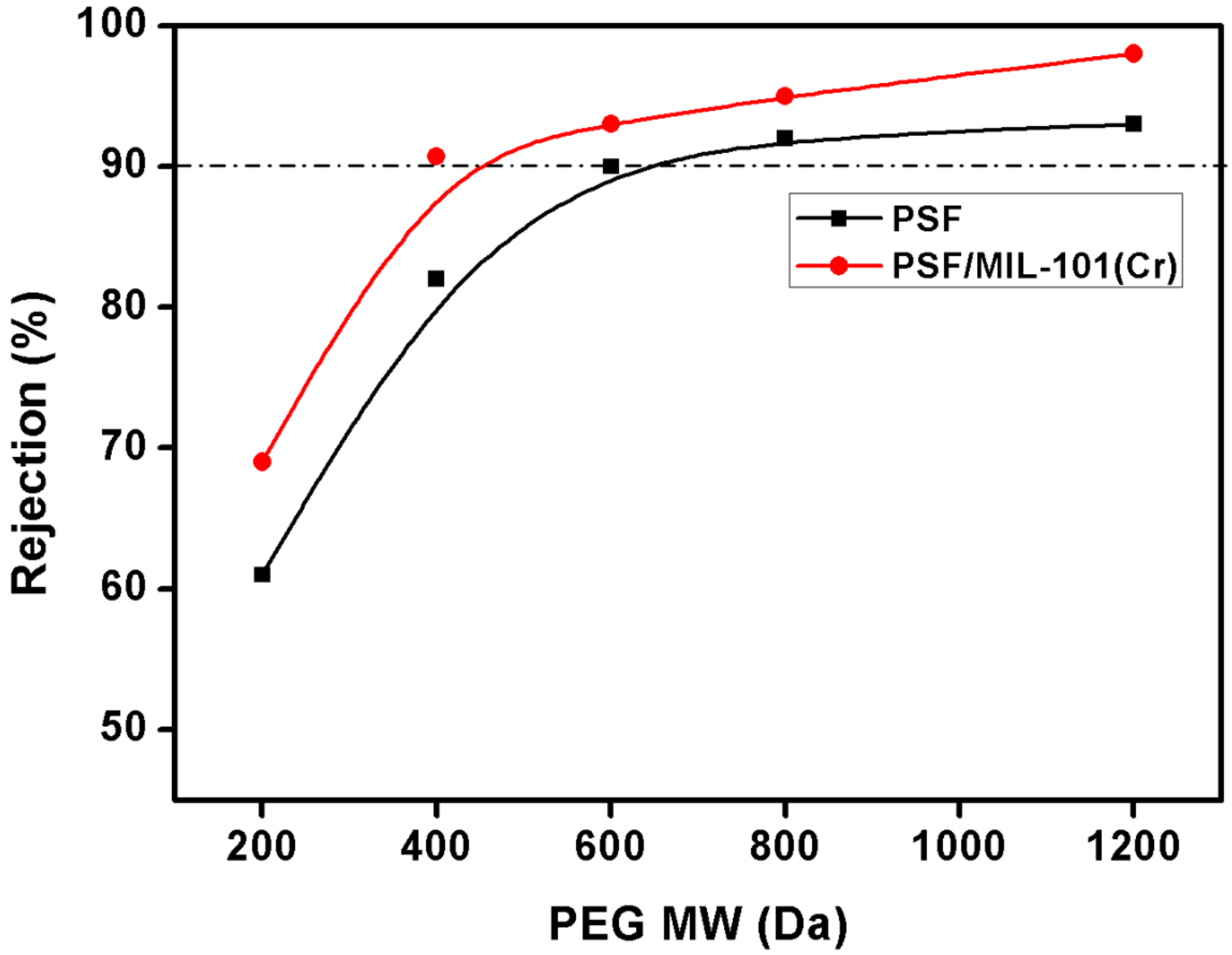

Figure S1. PEG rejection versus molecular weight (200–1200 Da) for pristine PSF and PSF/MIL-101(Cr) (0.75 wt%); the dashed line marks 90% rejection (MWCO)

<u>References</u>

[1] . Hou, Z. M., Tang, H., Hayder, H. A., Song, D. F., Li, M., Zuo, H. R., & Duan, M. (2026). Thermally stable polysulfone nanofiltration/reverse osmosis membranes via amino grafting. *Journal of Materials Chemistry A*, *14*(16), 9388-9409.

[2] Ma, D., Peh, S. B., Han, G., & Chen, S. B. (2017). Thin-film nanocomposite (TFN) membranes incorporated with super-hydrophilic metal–organic framework (MOF) UiO-66: toward enhancement of water flux and salt rejection. *ACS Applied Materials & Interfaces*, *9*(8), 7523-7534

[3] Ahmadipouya, S., Mousavi, S. A., Shokrgozar, A., & Mousavi, D. V. (2022). Improving dye removal and antifouling performance of polysulfone nanofiltration membranes by incorporation of UiO-66 metal-organic framework. *Journal of Environmental Chemical Engineering*, *10*(3), 107535.

[4] Mahdavi, H., Karami, M., & Heidari, A. A. (2021). Preparation of mixed matrix membranes made up of polysulfone and MIL-53 (Al) nanoparticles as promising membranes for separation of aqueous dye solutions. Separation and Purification Technology, 274, 119033.
